# Impact of Blood Pressure Across the Life Course on Arterial Stiffness in Midlife: The Mediating Role of Metabolic Factors

**DOI:** 10.1002/mco2.70440

**Published:** 2025-10-22

**Authors:** Yang Wang, Shi‐Qi Liu, Ze‐Jiaxin Niu, Ming‐Ke Chang, Ming‐Fei Du, Hao Jia, Yue Sun, Dan Wang, Gui‐Lin Hu, Zi‐Yue Man, Chao Chu, Teng Zhang, Xi Zhang, Yu Yan, Tong‐Shuai Guo, Rui‐Yu Wang, Sheng‐Hao Zuo, Hao Li, Lei Chen, Ying Xiong, Zhong‐Min Tian, Gregory Y. H. Lip, Zu‐Yi Yuan, Yu‐Ming Kang, Yao Lu, Jian‐Jun Mu

**Affiliations:** ^1^ Department of Cardiovascular Medicine First Affiliated Hospital of Xi'an Jiaotong University Xi'an China; ^2^ Clinical Research Center the Third Xiangya Hospital, Central South University Changsha China; ^3^ Department of Kidney Transplantation Hospital of Nephropathy, First Affiliated Hospital of Xi'an Jiaotong University Xi'an China; ^4^ Department of Critical Care Medicine First Affiliated Hospital of Xi'an Jiaotong University Xi'an China; ^5^ Department of Critical Care Nephrology and Blood Purification First Affiliated Hospital of Xi'an Jiaotong University Xi'an China; ^6^ Key Laboratory of Biomedical Information Engineering of Ministry of Education, School of Life Science and Technology, Xi'an Jiaotong University Xi'an China; ^7^ Liverpool Centre for Cardiovascular Science At University of Liverpool, Liverpool John Moores University, and Liverpool Heart & Chest Hospital Liverpool UK; ^8^ Department of Clinical Medicine Aalborg University Aalborg Denmark; ^9^ Medical University of Bialystok Bialystok Poland; ^10^ Department of Physiology and Pathophysiology Xi'an Jiaotong University School of Basic Medical Sciences, Shaanxi Engineering and Research Center of Vaccine, Key Laboratory of Environment and Genes Related to Diseases of Education Ministry of China Xi'an China

**Keywords:** arterial stiffness, blood pressure, childhood, mediation effect, triglyceride–glucose index

## Abstract

Childhood blood pressure (BP) is associated with increased arterial stiffness later in life. This study aimed to investigate the contributions of BP across different life stages to midlife arterial stiffness and the mediating role of metabolic factors. Using data from the Hanzhong Adolescent Hypertension Study, 1448 participants aged 6–18 years at baseline were prospectively followed for 30 years into adulthood. We used linear regression models to examine the associations between BP at different life stages and brachial‐ankle pulse wave velocity (baPWV). In addition, parallel multiple mediation analyses were conducted to evaluate the mediating roles of blood glucose and lipid metabolism in these associations. Significant associations between BP and adult baPWV were observed across childhood, adulthood, and cumulative long‐term BP burden, with BP in adulthood showing the strongest association. Additionally, the triglyceride–glucose index was identified as a mediator in the relationship between adult BP and midlife baPWV, with the mediation effects more pronounced among males. Our findings suggest that the detrimental impact of elevated BP on arterial stiffness begins early in life and intensifies over the lifespan, particularly during adulthood. Furthermore, the association between adult BP and arterial stiffness appears to be partially mediated by insulin resistance.

## Graphical abstract

This 30‐year cohort study demonstrates that elevated blood pressure (BP) since childhood contributes to arterial stiffness in midlife, with adult BP showing the strongest association. The triglyceride–glucose index partially mediates this relationship, especially in males. These findings highlight the importance of early BP control and metabolic health management to reduce the long‐term burden of arterial stiffness and associated cardiovascular risk.

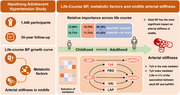

## 1 Introduction

Cardiovascular disease (CVD) remains the leading cause of mortality worldwide, posing a substantial burden on global health, particularly among the elderly population [1]. Arterial stiffness, a hallmark of vascular aging, has been strongly associated with an increased risk of CVD [2, 3]. Brachial‐ankle pulse wave velocity (baPWV) is a widely used, noninvasive indicator of arterial stiffness, with higher values indicating greater vascular rigidity [4]. Although clinical signs of arterial stiffness and atherosclerosis typically appear in middle or older age, the underlying pathological process of arterial stiffening often originates in childhood and progresses silently over decades [5]. Therefore, early identification and control of arterial stiffness and its modifiable risk factors are crucial to preventing future cardiovascular outcomes.

Previous studies have shown that elevated blood pressure (BP) not only accelerates end‐organ injury but also serves as a modifiable target for early intervention [6]. Emerging evidence suggests that childhood BP may influence arterial stiffness in adulthood, independent of BP levels later in life [7, 8, 9]. In addition, recent studies have highlighted the role of long‐term BP variability in vascular aging and arterial stiffening. For example, long‐term BP variability since childhood has been identified as a predictor of vascular aging in adulthood [10], and higher long‐term systolic BP (SBP) variability has been shown to independently contribute to the progression of arterial stiffness [11]. While these findings underscore the significance of BP dynamics across the life course, the relative contributions of BP at different life stages—childhood, adulthood, and cumulative long‐term burden—to arterial stiffness in midlife remain insufficiently understood.

Hypertension and metabolic dysfunction are both key contributors to arterial stiffness [12]. Metabolic dysfunction, including insulin resistance, obesity, and dyslipidemia, often coexists with elevated BP and may further accelerate vascular aging [13]. In addition, individuals with metabolic syndrome, insulin resistance, obesity, and dyslipidemia have an increased risk of hypertension [14, 15, 16, 17]. However, most studies have focused solely on the impact of metabolic dysfunction on hypertension, but the investigation of the reverse relationship that has explored the effects of hypertension or elevated BP on metabolic dysfunction was limited. Furthermore, to date, no studies have comprehensively investigated the potential mediating role of metabolic factors in the relationship between early‐life BP, cumulative BP burden, and arterial stiffness in midlife.

To address these gaps, we analyzed data from the Hanzhong Adolescent Hypertension Study, a longitudinal cohort that initially enrolled participants aged 6–18 years and followed them prospectively for 30 years into midlife [18, 19, 20, 21, 22]. This cohort is unique in China for comprehensively capturing the impact of the country's profound societal and economic transformations following the reform and opening‐up period on population health and disease risk. This study aimed to evaluate how BP measured at various stages across the life course contributes to arterial stiffness in midlife. In addition, we aimed to investigate the impact of elevated BP on metabolic dysfunction and to quantify the mediating effects of metabolic factors on the associations of childhood BP, adulthood BP, and cumulative lifetime BP burden with baPWV in adulthood.

## 2 Results

### 2.1 Characteristics of the Study Population

**FIGURE 1:**
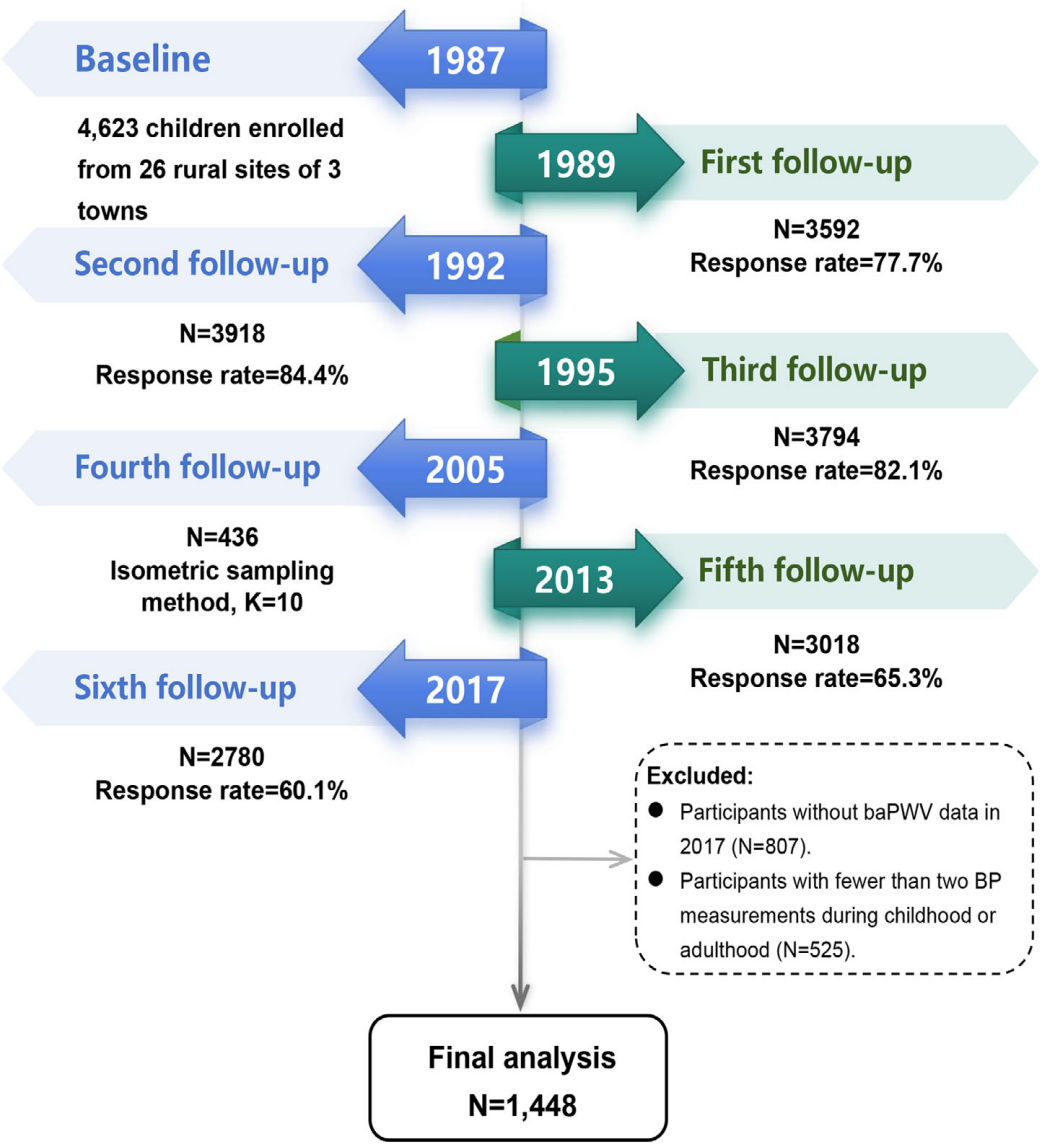
Flowchart of the study population.

This cohort study included 1448 participants who had undergone BP measurements at least four times from childhood to middle age and were retained for the final analyses (Figure 1). The demographic and clinical profiles of the participants are outlined in Table 1. Of the total cohort, 809 were male (55.9%) and 639 were female (44.1%), with a median age of 42 years.

**TABLE 1:** Demographic and clinical characteristics of study participants stratified by sex.

Variable	Total population	Males (*n* = 809)	Females (*n* = 639)	*p* Value
**Childhood (1987)**				
Age, years	12(9–14)	12(9–14)	12(10–14)	0.150
SBP, mmHg	102.0(96.0–110.0)	101.3(95.3–110.0)	102.7(96.7–110.0)	0.176
DBP, mmHg	64.0(60.0–70.7)	63.3(59.3–70.7)	64.7(60.0–70.7)	0.153
MAP, mmHg	71.1(71.5–83.3)	76.9(71.1–83.1)	77.5(72.3–83.3)	0.117
**Adulthood (2017)**				
Age, years	42(39–44)	42(39–44)	42(40–44)	0.150
Smoking, *n*(%)	627(44.4)	606(76.04)	21(3.42)	<0.001
Alcohol use, *n*(%)	416(29.5)	380(47.74)	36(5.86)	<0.001
Obesity, *n*(%)	209(14.4)	122(15.08)	87(13.62)	0.431
Physical activity				<0.001
Light, *n*(%)	85(5.00)	51(6.44)	34(5.55)	
Moderate, *n*(%)	745(53.10)	371(46.84)	374(61.11)	
Vigorous, *n*(%)	574(40.90)	370(46.72)	204(33.34)	
Diabetes, *n*(%)	310(21.97)	204(25.59)	106(17.26)	0.002
Hypertension, *n*(%)	240(17.01)	181(23.81)	59(9.61)	<0.001
SBP, mmHg	119.3(109.3–130.0)	123.3(115.3–132.7)	112.7(104.7–122.7)	<0.001
DBP, mmHg	79.3(71.3–86.7)	82.7(76.0–90.0)	75.3(68.7–80.7)	<0.001
MAP, mmHg	92.7(84.9–100.7)	96.4(89.8–103.3)	87.33(80.9–94.4)	<0.001
FBG, mmol/L	4.58(4.29–4.92)	4.60(4.29–4.93)	4.57(4.29–4.88)	0.134
LDL‐C, mmol/L	2.49(2.11–2.90)	2.56(2.18–3.01)	2.42(2.04–2.77)	<0.001
HDL‐C, mmol/L	1.14(0.99–1.33)	1.07(0.94–1.24)	1.24(1.08–1.43)	<0.001
TyG, mmol/L	6.92(6.57–7.34)	7.06(6.68–7.47)	6.73(6.44–7.13)	<0.001
LAP	30.10(17.89–52.27)	36.00(19.50–58.16)	25.00(16.68–41.19)	<0.001
VAI	1.79(1.17–2.77)	1.89(1.21–2.91)	1.65(1.13–2.57)	0.006
AIP	1.20(0.76–1.85)	1.45(0.94–2.20)	0.92(0.63–1.40)	<0.001
SUA, µmol/L	281.3(226.0–338.2)	322.9(281.2–370.8)	226.1(194.9–266.7)	<0.001
uACR, mg/g	8.57(5.53–15.14)	7.76(5.22–14.04)	9.73(6.14–17.40)	<0.001
baPWV, cm/s	1209.0(1092.8–1359.3)	1271.5(1153.5–1429.0)	1138.5(1030.0–1274.0)	<0.001
**Long‐term burden**				
SBP AUCt, mmHg	117.46(109.00–126.84)	121.68(113.40–130.57)	112.95(105.00–120.97)	0.149
DBP AUCt, mmHg	71.59(68.52–75.25)	71.73(68.67–75.41)	71.49(68.42–74.81)	0.159
MAP AUCt, mmHg	85.56(82.10–89.76)	85.72(82.23–90.05)	85.34(81.99–89.23)	0.139

Values are presented as median (interquartile range, IQR) for continuous variables (all non‐normally distributed) and *n* (%) for categorical variables.

Abbreviations: AIP, atherogenic index of plasma; AUCt, area under the curve for total burden; baPWV, brachial‐ankle pulse wave velocity. All results in the table were derived using the original, noninterpolated dataset; DBP, diastolic blood pressure; FBG, fasting blood glucose; HDL‐C, high‐density lipoprotein cholesterol; LAP, lipid accumulation product; LDL‐C, low‐density lipoprotein cholesterol; MAP, mean arterial pressure; SBP, systolic blood pressure; SUA, serum uric acid; TyG, triglyceride–glucose index; uACR, urinary albumin‐to‐creatinine ratio; VAI, visceral adiposity index.

Males exhibited a higher prevalence of smoking, alcohol consumption, vigorous physical activity, and greater waist circumference compared with females. In addition, males had significantly higher levels of metabolic markers, including low‐density lipoprotein cholesterol (LDL‐C), serum uric acid (SUA), visceral adiposity index (VAI), atherogenic index of plasma (AIP), and triglyceride–glucose (TyG) index, along with lower levels of high‐density lipoprotein cholesterol (HDL‐C). Males also demonstrated higher baPWV and adult BP compared with their female counterparts (Table 1). These findings suggest the presence of sex differences in baseline characteristics, highlighting the need for further analyses of sex‐specific differences in the impact of BP across the life course on midlife baPWV.

### 2.2 Associations Between BP Across the Life Course and BaPWV in Midlife

We used multiple linear regression models to investigate the relationships between BP measures at different life stages and adult baPWV. Childhood BP, adult BP, and cumulative BP burden (AUCt) were all significantly associated with adult baPWV after adjustment for potential confounders, including age, sex, obesity, smoking, alcohol use, physical activity, and either adult BP or BP AUCt (all *p *< 0.001; Table 2). Among these, adult BP showed the strongest association with baPWV [*β* = 7.88, 95% CI: 6.90–8.86 for SBP; *β* = 7.79, 95% CI: 6.86–8.72 for diastolic BP (DBP); *β* = 7.96, 95% CI: 7.07–8.84 for mean arterial pressure (MAP)], followed by childhood BP (*β* = 3.61, 95% CI: 2.53–4.69 for SBP; *β* = 2.89, 95% CI: 1.70–4.07 for DBP; *β* = 3.94, 95% CI: 2.66–5.22 for MAP), and BP AUCt (*β* = 2.78, 95% CI: 1.67–3.89 for SBP; *β* = 2.81, 95% CI: 1.01–4.61 for DBP; *β* = 2.47, 95% CI: 0.96–3.99 for MAP) (Table 2). In addition, to further validate our findings, we assessed the relative importance of BP measured at each life stage in relation to adult baPWV (Table 2). Of the total contribution, adulthood BP accounted for the largest proportion (SBP: 40.63%; DBP: 38.78%; MAP: 41.42%), followed by childhood BP and BP AUCt, which also made non‐negligible contributions (Table 2). These results suggest that although adult BP has the strongest impact on arterial stiffness, the influence of childhood BP and long‐term cumulative exposure should not be overlooked.

**TABLE 2:** Association between BP across the life course and adult baPWV.

		Outcome (baPWV)		
Life stage	Exposure (BP measures)	*β* (95%CI)	*p* Value	Relative importance
Childhood^a^	SBP	3.61 (2.53–4.69)	<0.001	16.73 %
	DBP	2.89 (1.70–4.07)	<0.001	12.60 %
	MAP	3.94 (2.66–5.22)	<0.001	15.70 %
Adulthood^b^	SBP	7.88 (6.90–8.86)	<0.001	40.63 %
	DBP	7.79 (6.86–8.72)	<0.001	38.78 %
	MAP	7.96 (7.07–8.84)	<0.001	41.42 %
AUCt^c^	SBP	2.78 (1.67–3.89)	<0.001	11.70 %
	DBP	2.81 (1.01–4.61)	0.002	10.76 %
	MAP	2.47 (0.96–3.99)	0.001	11.72 %

Relative importance was calculated using relative weight analysis, which decomposes the total *R* ^2^ from multiple regression models into weights that reflect the proportion of variance in the outcome variable (baPWV) attributable to each predictor (childhood BP, adulthood BP, and BP AUCt). This method accounts for multicollinearity among predictors by orthogonal transformation and is expressed as a percentage of the total explained variance.

Abbreviations: AUCt, area under the curve for total burden; baPWV, brachial‐ankle pulse wave velocity; BP, blood pressure; CI, confidence interval; DBP, diastolic blood pressure; MAP, mean arterial pressure; SBP, systolic blood pressure.

^a^ Adjusted for age, sex, obesity, smoking, alcohol use, and physical activity.

^b^ Adjusted for age, sex, obesity, smoking, alcohol use, physical activity, and BP AUCt.

^c^ Adjusted for age, sex, obesity, smoking, alcohol use, physical activity, and adulthood BP.

### 2.3 Mediation Effect of Metabolic Factors on the Association Between BP Across the Life Course and Adult BaPWV

We performed linear regression analyses to identify candidate mediators among glycolytic and lipid‐related metabolic markers, based on established evidence and biological plausibility (Figure S1). As shown in Table S1, adulthood BP was significantly associated with TyG index, fasting blood glucose (FBG), LAP, HDL, AIP, and VAI (*p *< 0.05). BP AUCt (cumulative burden) was significantly associated with TyG, LAP, AIP, and VAI (*p *< 0.05). However, no significant associations were observed between childhood BP and any of the metabolic markers (Table S1). In addition, among all those metabolic factors, TyG, FBG, LAP, and VAI were significantly associated with baPWV (Table S2). Therefore, only those metabolic markers that were significantly associated with both BP and baPWV were selected as potential mediators for further analysis. The final mediators included in this study were TyG, FBG, LAP, and VAI.

**FIGURE 2:**
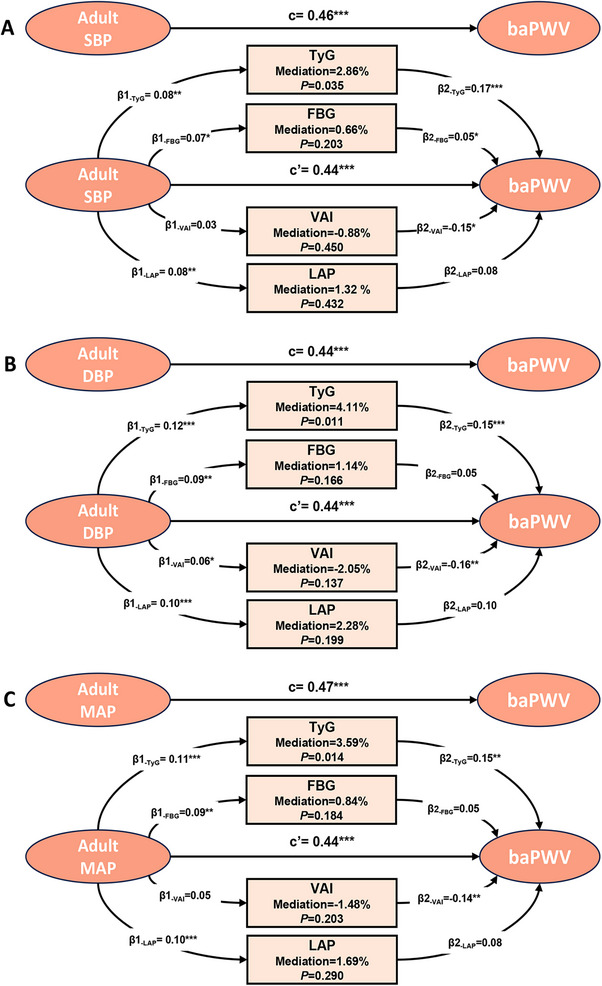
Mediation effects of multiple metabolic factors on the association between adult BP and midlife baPWV. BP: blood pressure; baPWV: brachial‐ankle pulse wave velocity; SBP: systolic blood pressure; DBP: diastolic blood pressure; MAP: mean arterial pressure; FBG: fasting blood glucose; TyG: index triglyceride–glucose index; VAI: visceral adiposity index. LAP: lipid accumulative index. Adjusted for age, sex, obesity, smoking, alcohol use, physical activity. *c*: Total effect of the predictor on the outcome; *β*_1_: effect of the predictor on the mediator; *β*_2_: effect of the mediator on the outcome (adjusting for the predictor); *c*′: direct effect of the predictor on the outcome (adjusting for the mediator). *p < 0.05; **p < 0.01; ***p < 0.001.

Next, we conducted multiple parallel mediation analyses to investigate the mediating role of metabolic factors in the relationship between BP across the life course and adult baPWV. The analyses revealed significant direct effects (*c*) of childhood BP, adult BP, total BP burden (AUCt) on adult baPWV (all *p* < 0.05). However, significant indirect effects were observed exclusively for adulthood BP, with no mediating effects detected for childhood BP or BP AUCt (Tables 3, S3, and S4). Among the metabolic factors, TyG index emerged as a significant mediator in the relationship between adult BP and adult baPWV. The indirect effects (*β*_Ind_) for SBP, DBP, and MAP were *β*_Ind_ = 0.013 (95% CI: 0.003–0.027), *β*_Ind_ = 0.018 (95% CI: 0.006–0.033), and *β*_Ind_ = 0.017(95% CI: 0.005–0.032), respectively (Table 3). The proportion of the mediation effects ranged from 2.86 to 4.11% (SBP: 2.86%, DBP: 4.11%, MAP: 3.59%) (Figure 2). These findings suggest that the association between adult BP and baPWV is partially mediated by the TyG index, reflecting the potential role of insulin resistance in the development of arterial stiffness.

**TABLE 3:** Mediation effects of metabolic factors on the relationship between adult BP and midlife baPWV.

Predictor	Mediator	*c* (SE)	*β*_1_ (SE)	*β*_2_ (SE)	*c*′ (95%CI)	*β*_Ind_ (95%CI)
SBP	TyG	0.455(0.025)^***^	0.079(0.028)^**^	0.171(0.044)^***^	0.437(0.381–0.494)^***^	0.013(0.003–0.027)^*^
	FBG	0.455(0.025)^***^	0.066(0.030)^*^	0.051(0.026)^*^	0.437(0.381–0.494)^***^	0.003(−0.001 to 0.010)
	VAI	0.455(0.025)^***^	0.030(0.029)	−0.146(0.054)^*^	0.437(0.381–0.494)^***^	−0.004(−0.018 to 0.003)
	LAP	0.455(0.025)^***^	0.076(0.027)^**^	0.078(0.062)	0.437(0.381–0.494)^***^	0.006(−0.004 to 0.020)
DBP	TyG	0.438(0.025)^***^	0.121(0.029)^***^	0.150(0.044)^***^	0.415(0.357–0.480)^***^	0.018(0.006–0.033)^*^
	FBG	0.438(0.025)^***^	0.094(0.027)^**^	0.050(0.026)	0.415(0.357–0.480)^***^	0.005(−0.000 to 0.012)
	VAI	0.438(0.025)^***^	0.059(0.027)^*^	−0.158(0.055)^**^	0.415(0.357–0.480)^***^	−0.009(−0.025 to −0.000)
	LAP	0.438(0.025)^***^	0.100(0.027)^***^	0.098(0.063)	0.415(0.357–0.480)^***^	0.010(−0.003 to 0.027)
MAP	TyG	0.474(0.025)^***^	0.111(0.028)^***^	0.154(0.043)^**^	0.453(0.392–0.515)^***^	0.017(0.005–0.032)^*^
	FBG	0.474(0.025)^***^	0.088(0.030)^**^	0.048(0.026)	0.453(0.392–0.515)^***^	0.004(−0.000 to 0.012)
	VAI	0.474(0.025)^***^	0.051(0.027)	−0.143(0.054)^**^	0.453(0.392–0.515)^***^	−0.007(−0.022 to 0.000)
	LAP	0.474(0.025)^***^	0.096(0.027)^***^	0.079(0.062)	0.453(0.392–0.515)^***^	0.008(−0.005 to 0.024)

*c*: Total effect of the predictor on the outcome; *β*_1_: effect of the predictor on the mediator; *β*_2_: effect of the mediator on the outcome (adjusting for the predictor); *c*′: direct effect of the predictor on the outcome (adjusting for the mediator); *β*_Ind_: indirect effect (mediation effect), calculated as *β*_1_ × *β*_2_. Adjusted for age, sex, obesity, smoking, alcohol use, and physical activity.

Abbreviations: baPWV, brachial‐ankle pulse wave velocity; BP, blood pressure; CI, confidence interval; DBP, diastolic blood pressure; FBG, fasting blood glucose; LAP, lipid accumulation product; MAP, mean arterial pressure; SBP, systolic blood pressure; SE, standard error; TyG, triglyceride–glucose index; VAI, visceral adiposity index.

* *p *< 0.05.

** *p *< 0.01.

*** *p *< 0.001.

### 2.4 Sex‐Specific Associations Between BP and Adult baPWV

**FIGURE 3:**
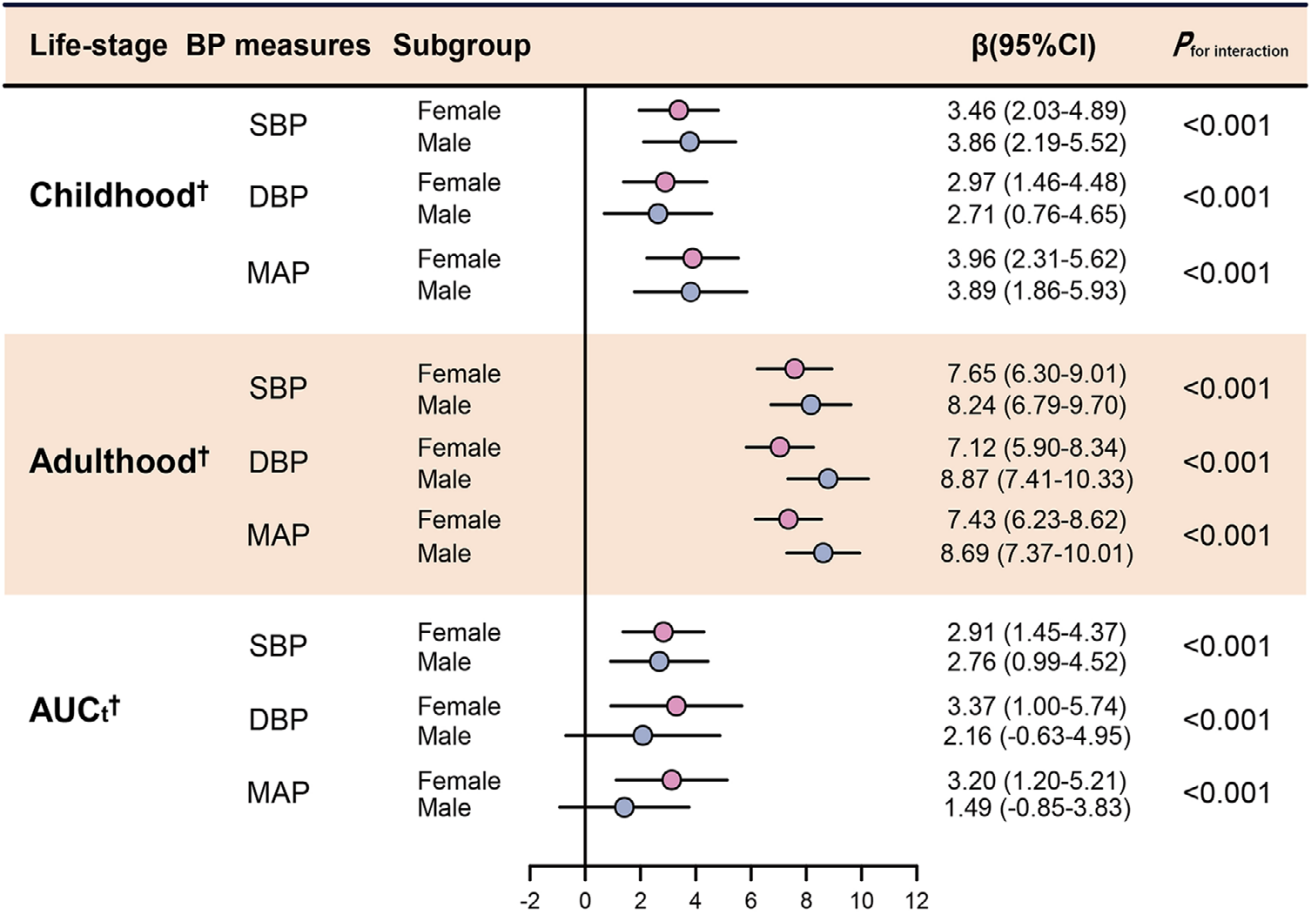
Sex‐specific analysis of the association between BP across the life course and adult baPWV. ^†^Adjusted for age, obesity, smoking, alcohol use, c activity. ^††^Adjusted for age, obesity, smoking, alcohol use, physical activity, and BP AUCt. ^†††^Adjusted for age, obesity, smoking, alcohol use, physical activity and adulthood BP. AUCt: area under the curve for total burden; baPWV: brachial‐ankle pulse wave velocity; BP: blood pressure.

**FIGURE 4:**
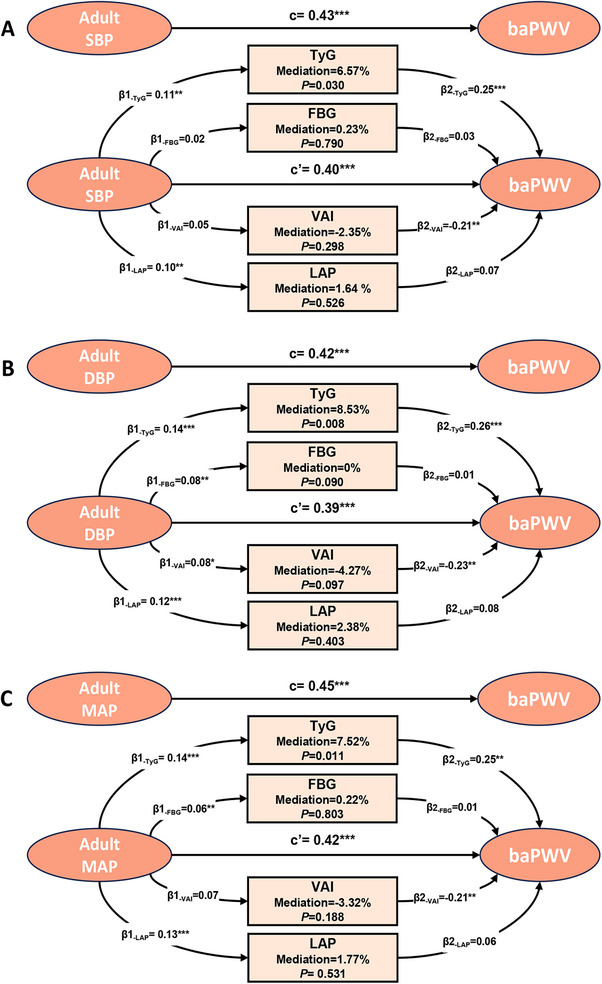
Mediation effects of multiple metabolic factors on the association between adult BP and midlife baPWV in males. BP, blood pressure; baPWV, brachial‐ankle pulse wave velocity; SBP, systolic blood pressure; DBP, diastolic blood pressure; MAP, mean arterial pressure; FBG, fasting blood glucose; TyG, triglyceride–glucose index; VAI, visceral adiposity index; LAP, lipid accumulation product. Adjusted for age, obesity, smoking, alcohol use, physical activity. *c*: Total effect of the predictor on the outcome; *β*_1_: effect of the predictor on the mediator; *β*_2_: effect of the mediator on the outcome (adjusting for the predictor); *c*′: direct effect of the predictor on the outcome (adjusting for the mediator). *p < 0.05; **p < 0.01; ***p < 0.001.

To assess sex‐specific differences, interaction terms were added to the multiple linear regression models. Significant interactions were observed for adult SBP, DBP, and MAP (*p* for interaction <0.05), indicating that the associations between adult BP and baPWV differ by sex (Figure 3). Sex‐stratified analyses further demonstrated that the associations between adult BP and baPWV were stronger in males than in females (Figure 3). In mediation analyses, the TyG index significantly mediated the association between adult BP and baPWV in males, with the proportion of mediation ranging from 6.57 to 8.53% (Figure 4 and Table S5). In contrast, no significant mediation effect was observed in females (Figure S2 and Table S6). These findings suggest that the impact of adult BP on midlife arterial stiffness is more pronounced in males and is partially mediated by insulin resistance, as reflected by the TyG index.

### 2.5 Sensitivity Analyses

We conducted several sensitivity analyses to confirm the robustness of our findings. First, we excluded participants with a self‐reported history of hypertension, diabetes, or cardiovascular events. The associations between BP and baPWV remained consistent (Tables S7 and S8). Second, we excluded individuals who reported using antihypertensive, hypoglycemic, or lipid‐lowering medications, and the results were similar (Tables S9 and S10). Third, to address the non‐normal distribution of baPWV, we applied a logarithmic transformation. The results remained consistent with those of the primary analysis (Tables S11 and S12). Finally, to account for potential bias due to missing data, we performed multiple imputation analyses under the assumption of missing at random. The imputed results were consistent with those from complete‐case analyses (Tables S13 and S14). Collectively, these sensitivity analyses confirm the robustness and reliability of our primary findings.

## 3 Discussion

This study provides novel insights into the life‐course association between BP and arterial stiffness in midlife, as well as the potential mediating role of metabolic factors in this relationship. First, we observed that BP levels at different life stages, including childhood, adulthood and long‐term BP burden, were significantly associated with midlife baPWV, a surrogate marker of arterial stiffness. Among these, adult BP exhibited the strongest association with baPWV; however, the contributions of childhood BP and total BP burden were also non‐negligible. Second, mediation analyses revealed that the TyG index partially mediated the association between adult BP and midlife arterial stiffness, underscoring the mechanistic role of insulin resistance. Collectively, these findings emphasize the need for early prevention and sustained BP monitoring throughout the life course, while also highlighting the importance of preserving metabolic health as an integral strategy for CVD prevention.

The development of arterial stiffness is a progressive and multifactorial process that originates early in life and advances with age. Previous studies have shown that childhood BP is a significant predictor of arterial stiffness in adulthood [7, 8, 9]. In addition, higher long‐term SBP variability has been independently associated with the progression of arterial stiffness, irrespective of average BP levels [10, 11]. Our prior studies further demonstrated that both the cumulative burden and life‐course trajectory of BP from childhood were significantly associated with increased baPWV in adulthood [23, 24]. However, the relative contributions of BP across different life stages to arterial stiffness remain unclear. Recent evidence from the Cardiovascular Risk in Young Finns Study indicated that mid‐adulthood SBP had the strongest association with arterial stiffness, whereas childhood and young adulthood SBP exerted comparatively weaker effects [25]. In line with these findings, the current study observed that although adult BP contributed most substantially to adult baPWV, the effects of childhood BP and cumulative BP burden were also significant and should not be overlooked. These findings highlight adulthood as a critical period for implementing preventive and therapeutic strategies to mitigate arterial stiffening. However, previous studies have demonstrated that BP tracks strongly from childhood into adulthood [26, 27]. Early‐life BP trajectories play a fundamental role in determining the risk of adult hypertension [28], and once elevated BP or hypertension is established, it becomes exceedingly difficult to reverse [29, 30]. This highlights the necessity of early, sustained BP monitoring and intervention across the life course to mitigate the gradual and often silent progression of arterial stiffness.

The association between elevated BP and arterial stiffness is well established; however, the potential mediating role of metabolic factors in this relationship—particularly across the long‐term transition from childhood to adulthood—remains insufficiently explored. The present study addresses this gap by identifying the TyG index as a mediator in the association between adult BP and midlife baPWV, thereby underscoring the role of insulin resistance in the pathophysiology of arterial stiffening. The TyG index is widely recognized as a reliable surrogate marker for insulin resistance, and its association with cardiovascular risk factors has been reported in clinical studies [31, 32]. Recent evidence further supports a robust association between elevated TyG levels and increased PWV [33, 34, 35]. Extending these findings, our analysis demonstrates that the TyG index partially mediates the relationship between adult BP and baPWV, suggesting that insulin resistance serves as an intermediary mechanism—beyond the direct hemodynamic burden of elevated BP—through which vascular stiffening progresses. These findings support a broader mechanistic framework linking metabolic dysfunction to vascular aging and highlight the potential utility of TyG as both a clinical marker and a therapeutic target.

In recent years, the relationship between insulin resistance and hypertension has garnered extensive attention. Insulin resistance is recognized as a central pathophysiological mechanism underpinning a range of chronic metabolic disorders and is increasingly implicated in the disruption of BP homeostasis [36]. Several studies have demonstrated a robust association between the TyG index and the risk of hypertension [15, 37, 38, 39]. For instance, a longitudinal cohort study reported that both baseline TyG levels and higher TyG trajectories were significantly associated with incident hypertension [15]. Similarly, a large‐scale population‐based study involving 47,808 participants identified strong associations between the TyG index and hypertension in both sexes [37]. While the contribution of insulin resistance to hypertension is well documented, evidence regarding the reverse pathway—namely, whether elevated BP may contribute to the development of insulin resistance—remains limited at the population level. Experimental studies suggest that sustained high BP may impair vascular endothelial integrity and insulin signaling, thereby promoting insulin resistance [40]. Specifically, hypertension‐induced endothelial dysfunction disrupts the insulin receptor substrate‐1 (IRS‐1)/PI3K/Akt signaling pathway, reducing glucose uptake in skeletal muscle and adipose tissue [40]. Moreover, diminished nitric oxide bioavailability due to endothelial injury compromises insulin‐mediated vasodilation, further aggravating insulin resistance [40, 41]. Inflammatory pathways and oxidative stress—driven by NADPH oxidase activation and proinflammatory cytokines such as TNF‐α and IL‐6—also contribute to insulin resistance by inducing serine phosphorylation of IRS‐1 and impairing insulin receptor activity [40, 41]. Our study reinforces the hypothesis that elevated BP may contribute to the development of insulin resistance, which in turn accelerates arterial stiffening. This bidirectional relationship underscores the need to consider metabolic dysfunction as both a consequence and a contributor to vascular pathology. Elucidating these mechanistic pathways offers critical insight for the design of early, multifaceted preventive strategies targeting both BP and metabolic regulation to mitigate long‐term cardiovascular risk.

An interesting finding of this study is the observed sex discrepancy in the mediating role of insulin resistance in the association between BP and arterial stiffness, with a more pronounced effect in males than females. The underlying mechanisms of this sex difference remain incompletely understood, though hormonal influences—particularly the vascular protective effects of estrogen—are likely contributors. Previous studies have shown that premenopausal women have a lower risk of atherosclerosis and coronary artery disease compared with age‐matched men, and this difference diminishes after menopause [42, 43]. Additionally, premenopausal women tend to exhibit lower PWV and pulse pressure than men; however, these measures increase substantially after menopause, often reaching or exceeding those observed in older men [44]. Estrogen has been shown to enhance endothelial function and reduce arterial stiffness, and estrogen therapy has been associated with vascular benefits in postmenopausal women [44]. Beyond hormonal factors, the renin–angiotensin–aldosterone system (RAAS) may also contribute to sex‐specific vascular differences. Experimental studies indicate that aldosterone receptor activation induces arterial stiffening and vascular fibrosis predominantly in male mice [45], whereas estrogen receptor‐α signaling may counteract these deleterious effects, potentially explaining the relative vascular resilience observed in females [46]. These findings suggest a complex interplay between sex hormones, RAAS activity, and metabolic regulation in determining vascular aging and stiffness. Further studies are needed to elucidate the mechanisms underlying these sex‐based differences and to guide the development of sex‐specific strategies for the prevention and management of CVD.

Our findings have important public health implications. First, by demonstrating that the detrimental effects of elevated BP on arterial stiffness originate in early life and progressively intensify over time, this study highlights the critical importance of lifelong BP monitoring and proactive management. Early identification of individuals at risk and timely intervention—especially among high‐risk populations—may substantially reduce the long‐term burden of arterial stiffness and its associated cardiovascular consequences. Second, this study offers novel insights into the complex interplay between BP, metabolic dysregulation, and vascular aging. The identification of the TyG index as a mediator in the BP–arterial stiffness pathway underscores the role of insulin resistance in this process. These findings suggest that integrated strategies addressing both BP control and metabolic health may be more effective in attenuating arterial stiffness and preventing downstream cardiovascular events. Third, the observed sex‐specific mediation effects, particularly the stronger influence of insulin resistance in males, indicate the necessity of incorporating sex‐specific factors into cardiovascular prevention and treatment strategies.

This study has several notable strengths. First, the large, prospective cohort captures the profound impact of societal and economic transformations on public health over the past three decades. Second, the long‐term follow‐up from childhood to middle age provides a unique opportunity to examine the life‐course impact of BP on arterial stiffness, offering valuable insights into the developmental origins of CVD. However, several limitations should be acknowledged. First, although our mediation model assumes a unidirectional pathway from BP to arterial stiffness, a potential bidirectional relationship cannot be entirely ruled out; arterial stiffness may also contribute to elevated BP through increased vascular resistance—an aspect not directly captured in our models. Second, the generalizability of the findings may be limited, as the study population consisted exclusively of Han Chinese participants. Third, although we focused on selected metabolic mediators, other biological pathways and unmeasured confounders may have influenced the associations observed. Fourth, although attrition over the long follow‐up period may introduce selection bias, comparisons of baseline characteristics between participants retained in the cohort and those lost to follow‐up showed no significant differences in key characteristics, indicating minimal bias and supporting the robustness of our findings.

## 4 Conclusion

This study identifies adulthood as a critical period during which elevated BP exerts the strongest influence on arterial stiffness in midlife. Mediation analyses further reveal the role of metabolic health—particularly insulin resistance, as indicated by the TyG index—in this association, with notable sex‐specific differences. These findings highlight the importance of adopting a life‐course approach to BP management that not only ensures effective BP control but also addresses underlying metabolic dysfunction to prevent arterial stiffening and its downstream cardiovascular consequences. Future research should aim to develop and evaluate targeted intervention strategies that integrate BP regulation with metabolic modulation to more effectively reduce long‐term cardiovascular risk.

## 5 Materials and Methods

### 5.1 Study Population

This study was conducted as part of the Hanzhong Adolescent Hypertension Study, a longitudinal, population‐based cohort designed to investigate the long‐term effects of early‐life cardiovascular and metabolic risk factors on health outcomes in midlife. The cohort was initiated in 1987, enrolling 4623 schoolchildren aged 6–18 years (mean age: 12.4 years; 56.5% male) from 26 rural communities across three towns in Hanzhong city, Shaanxi Province, China. Follow‐up visits were subsequently conducted in 1989, 1992, 1995, 2005, 2013, and 2017. Detailed descriptions of the cohort design and methodology have been reported elsewhere [18, 19, 20, 21, 22]. For the present analysis, of the 2780 participants followed up in 2017, individuals were excluded if they lacked baPWV measurements (*n* = 807) or had fewer than two BP measurements during either childhood or adulthood (*n* = 525). A total of 1448 participants were included in the final analysis. In this study, childhood BP was defined using measurements obtained in 1987, adulthood BP was derived from the 2013 visit, and baPWV measured in 2017 was used as the primary outcome. No significant differences in baseline characteristics were observed between participants included in the final analysis and those lost to follow‐up (Table S15).

### 5.2 Data Collection

Demographic information, including age, sex, smoking status, alcohol consumption, marital status, medical history, and physical activity levels, was collected through standardized questionnaires by trained personnel. Body weight and height were measured using calibrated instruments, and body mass index (BMI) was calculated as weight in kilograms divided by height in meters squared (kg/m^2^). BP was measured by trained and certified personnel using a standard mercury sphygmomanometer during most follow‐up visits, except in 2017, when an automated device (Omron HBP‐1100, Kyoto, Japan) was used, as previously described [10, 20]. Briefly, prior to BP measurements, participants were instructed to avoid consumption of coffee, tea, or alcohol, as well as smoking or engaging in vigorous physical activity, for at least 30 min. After resting in a seated position for 5 min, three BP measurements were obtained from the right upper arm, with a 2‐min interval between each reading. The average of the three values was used for subsequent analyses. MAP was calculated as: DBP + 1/3 × (SBP − DBP).

Fasting blood samples were collected during the 2017 follow‐up visit. Participants were instructed to abstain from food and beverages (except water) after dinner the night before, ensuring an overnight fast of at least 8 h. Blood was drawn in the morning (typically between 7:00 AM and 9:00 AM) to minimize diurnal variation in biochemical parameters. All samples were processed within 1–2 h to separate serum or plasma, then stored at −80°C until analysis. Biochemical indicators, including total cholesterol, triglycerides, HDL‐C, LDL‐C, TG, FBG, and SUA, were measured using an automated biochemical analyzer (Model 7600; Hitachi, Ltd., Tokyo, Japan) [19, 20, 21]. Details of the assay methods and definitions are provided in Supplementary I.

Insulin resistance was assessed using the TyG index, calculated as ln[TG(mg/dL) × FBG (mg/dL)/2]. The AIP was determined as the ratio of TG to HDL‐C (TG/HDL‐C). The VAI was calculated using sex‐specific formulas validated in the Chinese population: for males, VAI = [waist circumference/(39.68 + 1.88 × BMI)] × (TG/1.03) × (1.31/HDL‐C); for females, VAI = [waist circumference/(39.58 + 1.89 × BMI)] × (TG/0.81) × (1.52/HDL‐C). The LAP was calculated as (waist circumference − 65) × TG for males, and (waist circumference − 58) × TG for females [47, 48].

### 5.3 Long‐Term BP Exposure Calculation

To characterize the longitudinal patterns of BP over the life course, we calculated the area under the curve (AUC) based on individual BP growth curves (Figure S3) [49, 50]. The AUC approach provides a robust and integrative measure of cumulative BP exposure, accounting for both the magnitude and duration of BP levels across time. This method is particularly suitable for capturing the chronic effects of BP on vascular health, as it reflects total burden rather than isolated time‐point values [51]. BP growth curves were constructed using multiple BP measurements obtained from childhood through adulthood. These individual BP curves were modeled using Stata 17.0, incorporating both linear and nonlinear trends in BP progression over time. For each participant, the total AUC burden (AUCt) was computed as the integral of the BP growth curve across all time points, reflecting the cumulative BP load over the entire follow‐up period [7]. Compared with other longitudinal methods, such as linear mixed models or trajectory‐based clustering, the AUC method offers a more straightforward interpretation of cumulative BP effects and reduces potential biases arising from isolated BP fluctuations at single time points [52]. All AUC calculations were performed using validated statistical procedures to ensure the robustness and accuracy of long‐term BP exposure across the study population.

### 5.4 Evaluation of Arterial Stiffness

BaPWV is widely recognized as a noninvasive and reliable marker of arterial stiffness and has been extensively applied in clinical and epidemiological research [4]. In this study, baPWV was measured using a validated, automated waveform analyzer (BP‐203RPEII; Nihon Colin, Japan) following standardized measurement protocols [23, 53]. Briefly, cuffs were placed on both upper arms and ankles, while electrocardiogram electrodes were attached to the wrists to monitor cardiac rhythm. A phonocardiogram was used to detect heart sounds, allowing for accurate determination of pulse wave timing. The device automatically computed baPWV by dividing the transmission distance (estimated based on participant height) by the time interval between the upstroke of the brachial and ankle waveforms. Each participant underwent two consecutive baPWV assessments, and the mean of the bilateral values was used in the final analysis.

### 5.5 Statistical Analyses

The normality of all continuous variables was assessed using the Shapiro–Wilk test (Table S16). As all variables were non‐normally distributed, they are reported as medians (IQR), whereas categorical variables are expressed as counts and percentages (*n*, %). Sex‐based differences were evaluated using the Mann–Whitney *U* test for continuous variables and the chi‐square (*χ* ^2^) test for categorical variables. We performed multiple linear regression analyses to examine the associations between BP across the life course and arterial stiffness in adulthood. In these models, the outcome was adult baPWV. The independent variables included childhood BP, adult BP, and BP AUCt. All models were adjusted for potential confounders, including age, sex, smoking status, obesity, alcohol consumption, and physical activity in adulthood. Results are reported as standardized regression coefficients (*β*) with 95% confidence intervals (CIs). In the multicollinearity test, the variance inflation factor for each covariate was found to be less than 2, indicating no significant multicollinearity among the covariates (Table S17). To further quantify the relative contribution of each BP measure, we conducted relative weight analysis, which transforms correlated predictors into orthogonal components. The outcome was regressed on these uncorrelated components to obtain standardized coefficients (*β*_k_), and each predictor's relative weight was derived by combining these with the regression coefficients (*λ*_jk_) linking the original variables to the orthogonal components.

We conducted parallel multiple mediation analyses to investigate potential pathways through which childhood BP, adulthood BP, and BP AUCt affect adult baPWV. To align with our study objective, we assumed a unidirectional pathway from BP to arterial stiffness. Preliminary linear regression analyses were performed to identify candidate mediators by examining their associations with both the exposure variables (childhood BP, adulthood BP, and BP AUCt) and the outcome (adult baPWV). Only mediators significantly associated with both the exposures and the outcome were included in the final mediation models. The effect estimates (*β* coefficients) and corresponding *p* values are presented in Figure S4. All mediation models were adjusted for relevant confounders, including age, sex, obesity, smoking status, alcohol consumption, and physical activity, to reduce potential bias.

Parallel multiple mediation analyses were conducted following a four‐step analytical framework (Figure S5). (1) Total effect (*c*): the association between the predictor variable (*X*) and the outcome variable (*Y*) was assessed using the regression model: *Y* = *cX* + *e*, where *c* represents the total effect of *X* on *Y*. (2) Effect of *X* on mediator (*β*_1_): the impact of the predictor variable on the mediator (*M*) was examined using the model: *M* = *β*_1_ *X* + *e*, where *β*_1_ represents the effect of *X* on *M*. (3) Direct and indirect effects: To examine the mediator's influence on the outcome while adjusting for the predictor variable, the following model was applied: *Y* = *β*_2_ *M* + *c*′*X* + *e*, where *c*′ represents the direct effect of *X* on *Y*, and *β*_2_ indicates the effect of *M* on *Y*. The indirect effect was calculated as *β*_1_ × *β*_2_, quantifying the extent to which *X* influences *Y* through *M*. (4) Proportion of mediation effect: the proportion of the total effect mediated through M was calculated as: (*β*_1 _× *β*_2_/*c*) × 100%. In this analysis, the predictor variables (*X*) included childhood BP, adult BP, and BP AUCt; the mediator variables (*M*) were the TyG index, FBG, LAP, and VAI; and the outcome variable (*Y*) was adult baPWV. To ensure robust estimation, we applied bootstrap sampling with 1000 iterations to generate percentile‐based 95% CIs for the indirect effect (*β*_Ind _= *β*_1 _× *β*_2_), the direct effect (*c*′), and the proportion of mediation effect (*β*_1_ × *β*_2_/*c*). A mediation effect was considered statistically significant if the 95% CI of the indirect effect did not include zero. All statistical analyses were conducted using R software (version 4.2.1), with a two‐sided *p* value <0.05 considered statistically significant.

## Author Contributions

Yang Wang and Yao Lu conceived and designed the study. Jian‐Jun Mu and Ying Xiong oversaw participant recruitment. Ze‐Jiaxin Niu, Ming‐Ke Chang, Ming‐Fei Du, Hao Jia, Yue Sun, Dan Wang, Gui‐Lin Hu, Zi‐Yue Man, Chao Chu, Teng Zhang, Xi Zhang, Yu Yan, Tong‐Shuai Guo, Rui‐Yu Wang, and Sheng‐Hao Zuo contributed to participant follow‐up and data collection. Yang Wang, Shi‐Qi Liu, and Ze‐Jiaxin Niu conducted the data analyses and drafted the manuscript. Hao Li, Lei Chen, Zhong‐Min Tian, Gregory Y. H. Lip, Zu‐Yi Yuan, Yu‐Ming Kang, and Jian‐Jun Mu critically reviewed and revised the manuscript. All authors read and approved the final version of the manuscript.

## Ethics Statements

The study protocol was approved by the Ethics Committee of the First Affiliated Hospital of Xi'an Jiaotong University (XJTU1AF2015LSL‐047). Written informed consent was obtained from all participants at each follow‐up visit. For individuals under 18 years of age, consent was obtained from a parent or legal guardian. All study procedures adhered to institutional guidelines and were conducted in accordance with the principles outlined in the Declaration of Helsinki. This study is registered with ClinicalTrials.gov (Identifier: NCT02734472).

## Conflicts of Interest

The authors declare no conflicts of interest.

## Supporting information




**Supplementary I**: Definition of clinical characteristics.
**Table S1**: Association between BP across the life course and multiple metabolic factors.
**Table S2**: Association between adult baPWV and multiple metabolic factors.
**Table S3**: Mediation effects of metabolic factors on the relationship between childhood BP and midlife baPWV.
**Table S4**: Mediation effects of metabolic factors on the relationship between BP AUCt and midlife baPWV.
**Table S5**: Mediation effects of multiple metabolic factors on the relationship between adult BP and midlife baPWV in males.
**Table S6**: Mediation effects of multiple metabolic factors on the relationship between adult BP and midlife baPWV in females.
**Table S7**: Association between BP across the life course and adult baPWV among participants without hypertension, diabetes, and cardiovascular events (*n* = 1333).
**Table S8**: Mediation effects of multiple metabolic factors on the relationship between adult BP and midlife baPWV among participants without hypertension, diabetes and cardiovascular events (*n* = 1333).
**Table S9**: Association between BP across the life course and adult baPWV among participants without medication use (*n* = 1307).
**Table S10**: Mediation effects of multiple metabolic factors on the relationship between adult BP and midlife baPWV among participants without medication use (*n* = 1307).
**Table S11**: Association between BP across the life course and adult log‐transformed baPWV.
**Table S12**: Mediation effects of multiple metabolic factors on the relationship between adult BP and log‐transformed baPWV in midlife.
**Table S13**: Association between BP across the life course and adult baPWV among participants with complete data after multiple imputation.
**Table S14**: Mediation effects of multiple metabolic factors on the relationship between adult BP and midlife baPWV among participants with complete data after multiple imputation.
**Table S15**: Comparison of baseline demographic and clinical characteristics between participants who remained in the final analysis and those who dropped out during follow‐up.
**Table S16**: Normality assessment of continuous variables using the Shapiro–Wilk test.
**Table S17**: Assessment of collinearity among covariates.
**Figure S1**: Potential Biological mechanisms underlying the relationships among hypertension, obesity, dyslipidemia, insulin resistance and arterial stiffness. RAAS, renin–angiotensin–aldosterone system; SNS, sympathetic nervous system; EVs, extracellular vesicles; SGLT2, sodium‐glucose co‐transporter 2.
**Figure S2**: Mediation effects of multiple metabolic factors on the association between adult BP and midlife baPWV in females. BP, blood pressure; baPWV, brachial‐ankle pulse wave velocity; SBP, systolic blood pressure; DBP, diastolic blood pressure; MAP, mean arterial pressure; FBG, fasting blood glucose; TyG, index triglyceride–glucose index; VAI, visceral adiposity index. LAP, lipid accumulation product. Adjusted for age, obesity, smoking, alcohol use, physical activity. *c*: total effect of the predictor on the outcome; *β*_1_: effect of the predictor on the mediator; *β*_2_: effect of the mediator on the outcome (adjusting for the predictor); *c*′: direct effect of the predictor on the outcome (adjusting for the mediator). *p < 0.05; **p < 0.01; ***p < 0.001.
**Figure S3**: Schematic diagram illustrating the calculation of long‐term cumulative blood pressure exposure using the area under the curve (AUC). BP, blood pressure. AUC, area under the curve.
**Figure S4**: Correlations among multiple metabolic factors. AIP, atherosclerotic index of plasma; HDL‐C, high‐density lipoprotein cholesterol; LAP, lipid accumulation product; LDLC, low‐density lipoprotein cholesterol; FBG, fasting blood glucose; TyG, triglyceride–glucose index; VAI, visceral adiposity index.
**Figure S5**: Schematic representation of the framework for multiple parallel mediation analysis. A: the framework of multiple parallel mediation; B: calculation of the proportion of mediation effects.

## Data Availability

The datasets used and analyzed during the current study are available from the corresponding authors upon reasonable request, without restriction, after publication.
