# Supplementary material for: Impact of Blood Pressure Across the Life Course on Arterial Stiffness in Midlife: The Mediating Role of Metabolic Factors
Source: MedComm (2020). 2025 Oct 22;6(11):e70440. doi: 10.1002/mco2.70440 (PMC12547078; doi:10.1002/mco2.70440)
Supplement: Supplementary file 1 — Supplementary I: Definition of clinical characteristics. Table S1: Association between BP across the life course and multiple metabolic factors. Table S2: Association between adult baPWV and multiple metabolic factors. Table S3: Mediation effects of metabolic factors on the relationship between childhood BP and midlife baPWV. Table S4: Mediation effects of metabolic factors on the relationship between BP AUCt and midlife baPWV. Table S5: Mediation effects of multiple metabolic factors on the relationship between adult BP and midlife baPWV in males. Table S6: Mediation effects of multiple metabolic factors on the relationship between adult BP and midlife baPWV in females. Table S7: Association between BP across the life course and adult baPWV among participants without hypertension, diabetes, and cardiovascular events (n = 1333). Table S8: Mediation effects of multiple metabolic factors on the relationship between adult BP and midlife baPWV among participants without hypertension, diabetes and cardiovascular events (n = 1333). Table S9: Association between BP across the life course and adult baPWV among participants without medication use (n = 1307). Table S10: Mediation effects of multiple metabolic factors on the relationship between adult BP and midlife baPWV among participants without medication use (n = 1307). Table S11: Association between BP across the life course and adult log‐transformed baPWV. Table S12: Mediation effects of multiple metabolic factors on the relationship between adult BP and log‐transformed baPWV in midlife. Table S13: Association between BP across the life course and adult baPWV among participants with complete data after multiple imputation. Table S14: Mediation effects of multiple metabolic factors on the relationship between adult BP and midlife baPWV among participants with complete data after multiple imputation. Table S15: Comparison of baseline demographic and clinical characteristics between participants who remained in the final a [file MCO2-6-e70440-s001.pdf]

Impact of Blood Pressure Across the Life Course on Arterial Stiffness in Midlife: The Mediating Role of Metabolic Factors

Yang Wang[1#], Shi-Qi Liu[2#], Ze-Jiaxin Niu[3#], Ming-Ke Chang[1], Ming-Fei Du[1], Hao Jia[1], Yue Sun[1], Dan Wang[1], Gui-Lin Hu[1], Zi-Yue Man[1], Chao Chu[1], Teng Zhang[1], Xi Zhang[1], Yu Yan[1], Tong-Shuai Guo[1], Rui-Yu Wang[1], Sheng-Hao Zuo[1], Hao Li[4], Lei Chen[5], Ying Xiong[1], Zhong-Min Tian[6], Gregory Y. H. Lip[7,8,9], Zu-Yi Yuan[1], Yu-Ming Kang[10*], Yao Lu[2*], Jian-Jun Mu[1*]

1. Department of Cardiovascular Medicine, First Affiliated Hospital of Xi'an Jiaotong University, Xi'an, China;
2. Clinical Research Center, the Third Xiangya Hospital, Central South University, Changsha, China;
3. Department of Kidney Transplantation, Hospital of Nephropathy, The First Affiliated Hospital of Xi'an Jiaotong University, Xi'an, China;
4. Department of Critical Care Medicine, First Affiliated Hospital of Xi'an Jiaotong University, Xi'an, China;
5. Department of Critical Care Nephrology and Blood Purification, First Affiliated Hospital of Xi'an Jiaotong University, Xi'an, China;
6. Key Laboratory of Biomedical Information Engineering of Ministry of Education, School of Life Science and Technology, Xi'an Jiaotong University, Xi'an, China;
7. Liverpool Centre for Cardiovascular Science at University of Liverpool, Liverpool John Moores University, and Liverpool Heart & Chest Hospital, Liverpool, United Kingdom;
8. Department of Clinical Medicine, Aalborg University, Aalborg, Denmark;
9. Medical University of Bialystok, Bialystok, Poland;
10. Department of Physiology and Pathophysiology, Xi'an Jiaotong University School of Basic Medical Sciences, Shaanxi Engineering and Research Center of Vaccine, Key

Laboratory of Environment and Genes Related to Diseases of Education Ministry of China, China.

[#] These authors contributed equally to this work.

*** Corresponding authors:**

Prof. Yu-Ming Kang, Department of Physiology and Pathophysiology, Xi’an Jiaotong University School of Basic Medical Sciences, 76 Yanta West Road, Xi’an, 710061, China. E-mail: ykang@mail.xjtu.edu.cn.

or

Prof. Yao Lu, Clinical Research Center, the Third Xiangya Hospital, Central South University, 138 Tongzipo Road, Changsha, 410013, China. E-mail: luyao0719@csu.edu.cn.

or

Prof. Jian-Jun Mu, Department of Cardiovascular Medicine, First Affiliated Hospital of Xian Jiaotong University, 277 Yanta West Road, Xi’an, 710061, China. E-mail: mujjun@mail.xjtu.edu.cn.

Supplementary I. Definition of clinical characteristics

Blood biochemical analyses

Blood samples were obtained via peripheral venipuncture, immediately centrifuged at 3,000 × *g* for 10 minutes, and stored at −80°C until analysis. Levels of total cholesterol (TC), triglycerides (TG), high-density lipoprotein cholesterol (HDL-C), low-density lipoprotein cholesterol (LDL-C), serum creatinine, and blood glucose were measured using a fully automated biochemical analyzer (Model 7600; Hitachi, Ltd., Tokyo, Japan). Serum uric acid (SUA) was measured using the uricase HMMPS method on the same analyzer. The intra- and inter-assay coefficients of variation (CV), assessed using five control samples, ranged from 2.3% to 4.5% and 3.2% to 6.4%, respectively. All assays were performed in the Department of Laboratory Medicine at the First Affiliated Hospital of Xi'an Jiaotong University.

Urinary biochemical analyses

A morning fasting midstream urine sample was collected from each participant and stored at −20 °C to −40 °C. All samples were transported in iceboxes under ambient packaging conditions to the clinical laboratory at the First Affiliated Hospital of Xi'an Jiaotong University (Xi'an, China). Urinary creatinine and albumin concentrations were measured using an automated biochemical analyzer (Hitachi, Ltd., Japan) in a certified clinical laboratory. The intra- and inter-assay coefficients of variation (CVs) were 1.25% and 2.17% for urinary creatinine, and 0.64% and 2.13% for urinary albumin, respectively.

Definitions

Participants who reported continuous or cumulative cigarette smoking for six months or more during their lifetime were classified as smokers. Alcohol consumption was defined as the daily intake of liquor, beer, or wine for at least six months. Marital status was categorized as

single, married, divorced, or widowed. Physical inactivity was defined as engaging in mild to moderate physical activity for less than three hours per week. Hypertension was defined as a systolic blood pressure (SBP) ≥ 140 mmHg, diastolic blood pressure (DBP) ≥ 90 mmHg, or the use of antihypertensive medications, based on clinical measurements or self-reported history. Diabetes was defined as a fasting blood glucose level ≥ 7.0 mmol/L, current use of antidiabetic medications, or a previously confirmed diagnosis of diabetes mellitus. Obesity in adulthood was defined as a body mass index (BMI) ≥ 30.0 kg/m^2.

Table S1. Association between BP across the life course and multiple metabolic factors.

Life-stage	Metabolic factors	SBP		DBP		MAP	
		**β (95%CI)**	***P* value**	**β (95%CI)**	***P* value**	**β (95%CI)**	***P* value**
Childhood[†]	TyG	0.001(-0.002–0.004)	0.418	0.002(-0.002–0.005)	0.335	0.002(-0.001–0.006)	0.250
	FBG	0.007(0.000–0.014)	0.061	0.000(-0.007–0.008)	0.898	0.003(-0.005–0.011)	0.441
	LDL-C	-0.002(-0.005–0.002)	0.378	-0.004(-0.008–0.000)	0.064	-0.004(-0.008–0.000)	0.067
	LAP	0.174(-0.022–0.370)	0.081	0.097(-0.116–0.311)	0.372	0.161(-0.071–0.392)	0.173
	HDL-C	0.000(-0.001–0.001)	0.890	-0.001(-0.002–0.001)	0.197	-0.001(-0.002–0.001)	0.295
	AIP	0.002(-0.007–0.011)	0.598	0.005(-0.005–0.014)	0.366	0.005(-0.005–0.016)	0.340
	VAI	0.005(-0.008–0.018)	0.484	0.007(-0.008–0.021)	0.360	0.008(-0.007–0.023)	0.311
Adulthood[††]	TyG	0.006(0.002–0.009)	<0.001	0.006(0.003–0.009)	<0.001	0.005(0.002–0.008)	<0.001
	FBG	0.009(0.002–0.016)	0.014	0.010(0.003–0.016)	0.003	0.010(0.003–0.016)	0.009
	LDL-C	-0.001(-0.004–0.003)	0.745	0.003(0.000–0.006)	0.075	0.002(-0.001–0.006)	0.126
	LAP	0.399(0.204–0.594)	<0.001	0.327(0.142–0.511)	0.001	0.288(0.110–0.466)	0.002
	HDL-C	-0.002(-0.003–-0.001)	0.003	-0.002(-0.003–0.000)	0.011	-0.002(-0.003–0.000)	0.007
	AIP	0.013(0.004–0.021)	0.006	0.008(0.000–0.017)	0.055	0.007(-0.002–0.015)	0.119
	VAI	0.019(0.006–0.032)	0.005	0.011(-0.001–0.024)	0.074	0.009(-0.003–0.020)	0.158
AUCt[†††]	TyG	-0.004(-0.008–-0.001)	0.018	0.000(-0.005–0.006)	0.942	0.001(-0.004–0.006)	0.718
	FBG	-0.006(-0.014–0.001)	0.107	-0.001(-0.014–0.011)	0.817	0.001(-0.010–0.012)	0.872
	LDL-C	0.002(-0.001–0.006)	0.223	0.001(-0.005–0.007)	0.745	0.001(-0.005–0.006)	0.781
	LAP	-0.381(-0.603–-0.159)	0.001	-0.045(-0.403–0.312)	0.803	-0.016(-0.323–0.292)	0.921

HDL-C	0.001(0.000–0.003)	0.156	-0.001(-0.003–0.002)	0.678	-0.001(-0.003–0.002)	0.604
AIP	-0.016(-0.026—0.005)	0.003	-0.003(-0.020–0.013)	0.711	-0.001(-0.016–0.013)	0.842
VAI	-0.025(-0.040—0.010)	0.001	-0.007(-0.030–0.017)	0.589	-0.004(-0.024–0.017)	0.709

†Adjusted for age, sex, obesity, smoking, alcohol use, and physical activity. ††Adjusted for age, sex, obesity, smoking, alcohol use, physical activity and BP AUCt. †††Adjusted for age, sex, obesity, smoking, alcohol use, physical activity and adulthood BP. AUCt, area under the curve for total burden; BP, blood pressure; SBP, systolic blood pressure; DBP, diastolic blood pressure; MAP, mean arterial pressure. FBG, fasting blood glucose; LDL-C, low-density lipoprotein cholesterol; HDL-C, high-density lipoprotein cholesterol; TyG, triglyceride–glucose index; LAP, lipid accumulation product; VAI, visceral adiposity index; AIP, atherogenic index of plasma; CI, confidence interval.

Table S2. Association between adult baPWV and multiple metabolic factors.

**Metabolic factors**	**baPWV**	
	**β (95%CI)**	***P* value**
TyG	46.045(29.237–62.853)	<0.001
FBG	16.905(9.467–24.343)	<0.001
LDL-C	12.398(-2.623–27.420)	0.106
LAP	0.433(0.169–0.697)	0.001
HDL-C	8.142(-29.780–46.064)	0.674
AIP	5.205(-0.557–10.968)	0.077
VAI	4.017(0.060–7.974)	0.047

Adjusted for age, sex, obesity, smoking, alcohol use, physical activity, diabetes, and hypertension. BaPWV, brachial-ankle pulse wave velocity; FBG, fasting blood glucose; LDL-C, low-density lipoprotein cholesterol; HDL-C, high-density lipoprotein cholesterol; TyG, triglyceride–glucose index; LAP, lipid accumulation product; VAI, visceral adiposity index; AIP, atherogenic index of plasma; CI, confidence interval.

Table S3. Mediation effects of metabolic factors on the relationship between childhood BP and midlife baPWV.

Predictor	Mediator	c (SE)	β1 (SE)	β2 (SE)	c' (95%CI)	β_{Ind} (95%CI)
SBP	TyG	0.161(0.026)***	0.024(0.027)	0.199(0.048)***	0.152(0.102–0.204)***	0.005(-0.005–0.016)
	FBG	0.161(0.026)***	0.044(0.028)	0.059(0.029)*	0.152(0.102–0.204)***	0.003 (-0.001–0.008)
	VAI	0.161(0.026)***	0.024(0.028)	-0.228(0.059)***	0.152(0.102–0.204)***	-0.005(-0.023–0.006)
	LAP	0.161(0.026)***	0.046(0.026)	0.158(0.068)*	0.152(0.102–0.204)***	0.007(-0.001–0.022)
DBP	TyG	0.114(0.026)***	0.032(0.026)	0.186 (0.048)***	0.111(0.059–0.164)***	0.006(-0.003–0.017)
	FBG	0.114(0.026)***	0.001(0.027)	0.068 (0.029)*	0.111(0.059–0.164)***	0.000(-0.004–0.003)
	VAI	0.114(0.026)***	0.032(0.027)	-0.247(0.059)**	0.111(0.059–0.164)***	-0.008(-0.020–0.002)
	LAP	0.114(0.026)***	0.026(0.025)	0.189(0.068)**	0.111(0.059–0.164)***	0.005(-0.004–0.017)
MAP	TyG	0.146(0.026)***	0.036(0.026)	0.188(0.048)**	0.140(0.089–0.193)***	0.007(-0.003–0.018)
	FBG	0.146(0.026)***	0.016(0.028)	0.066(0.029)*	0.140(0.089–0.193)***	0.001(-0.002–0.005)
	VAI	0.146(0.026)***	0.035(0.028)	-0.242(0.059)***	0.140(0.089–0.193)***	-0.008(-0.021–0.001)
	LAP	0.146(0.026)***	0.037(0.026)	0.180(0.068)**	0.140(0.089–0.193)***	0.007(-0.001–0.019)

c: total effect of the predictor on the outcome; β_1: effect of the predictor on the mediator; β_2: effect of the mediator on the outcome (adjusting for the predictor); c′: direct effect of the predictor on the outcome (adjusting for the mediator); β_{Ind}: indirect effect (mediation effect), calculated as $\beta_1 \times \beta_2$. Adjusted for age, sex, obesity, smoking, alcohol use, and physical activity. BP, blood pressure; baPWV, brachial-ankle pulse wave velocity; SBP, systolic blood pressure; DBP, diastolic blood pressure; MAP, mean arterial pressure; TyG, triglyceride-glucose index; FBG, fasting blood glucose; VAI, visceral adiposity index; LAP, lipid accumulation product; SE, standard error; CI, confidence interval. $^{*}P<0.05$; $^{**}P<0.01$; $^{***}P<0.001$.

Table S4. Mediation effects of metabolic factors on the relationship between BP AUCt and midlife baPWV.

Predictor	Mediator	c (SE)	β1 (SE)	β2 (SE)	c' (95%CI)	β_{Ind} (95%CI)
SBP	TyG	0.262(0.026)***	0.018(0.028)	0.222(0.042)***	0.251(0.197–0.303)***	0.004(-0.008–0.018)
	FBG	0.262(0.026)***	-0.036(0.029)	-0.196(0.058)***	0.251(0.197–0.303)***	0.007(-0.006–0.020)
	VAI	0.262(0.026)***	0.003 (0.027)	0.131(0.066)*	0.251(0.197–0.303)***	0.000(-0.008–0.011)
	LAP	0.262(0.026)***	0.018(0.028)	0.222(0.042)***	0.251(0.197–0.303)***	0.004(-0.008–0.018)
DBP	TyG	0.102(0.026)***	0.010(0.026)	0.185(0.048)***	0.111(0.059–0.164)***	0.002(-0.008–-0.011)
	FBG	0.102(0.026)***	0.005(0.028)	0.075(0.029)**	0.111(0.059–0.164)***	0.000(-0.003–0.005)
	VAI	0.102(0.026)***	-0.008(0.027)	-0.239(0.060)***	0.111(0.059–0.164)***	0.002(-0.011–0.015)
	LAP	0.102(0.026)***	0.002(0.026)	0.180(0.069)**	0.111(0.059–0.164)***	0.000(-0.008–0.012)
MAP	TyG	0.109(0.026)***	0.017(0.026)	0.185(0.048)***	0.103(0.052–0.154)***	0.003(-0.006–0.013)
	FBG	0.109(0.026)***	0.017(0.028)	0.073(0.029)*	0.103(0.052–0.154)***	0.001(-0.001–0.006)
	VAI	0.109(0.026)***	-0.004(0.027)	-0.239(0.060)***	0.103(0.052–0.154)***	0.001(-0.011–0.012)
	LAP	0.109(0.026)***	0.007(0.026)	0.180(0.069)**	0.103(0.052–0.154)***	0.001(-0.007–0.013)

c: total effect of the predictor on the outcome; β_1: effect of the predictor on the mediator; β_2: effect of the mediator on the outcome (adjusting for the predictor); c′: direct effect of the predictor on the outcome (adjusting for the mediator); β_{Ind}: indirect effect (mediation effect), calculated as $\beta_1 \times \beta_2$. Adjusted for age, sex, obesity, smoking, alcohol use, and physical activity. BP, blood pressure; AUCt, area under the curve for total burden; baPWV, brachial-ankle pulse wave velocity; SBP, systolic blood pressure; DBP, diastolic blood pressure; MAP, mean arterial pressure; TyG, triglyceride-glucose index; FBG, fasting blood glucose; VAI, visceral adiposity index; LAP, lipid accumulation product; SE, standard error; CI, confidence interval. $^{*}P<0.05$; $^{**}P<0.01$; $^{***}P<0.001$.

Table S5. Mediation effects of multiple metabolic factors on the relationship between adult BP and midlife baPWV in males.

**Predictor**	**Mediator**	**c (SE)**	**β1 (SE)**	**β2 (SE)**	**c' (95%CI)**	**β_{Ind} (95%CI)**
SBP	TyG	0.426(0.033)***	0.110 (0.036)**	0.252(0.061)***	0.400(0.343–0.474)***	0.069(0.009–0.058)*
	FBG	0.426(0.033)***	0.023(0.037)	0.026(0.037)	0.400(0.343–0.474)***	0.001(-0.004–0.006)
	VAI	0.426(0.033)***	0.048(0.037)	-0.211(0.076)**	0.400(0.343–0.474)***	-0.010(-0.044–0.003)
	LAP	0.426(0.033)***	0.104(0.035)**	0.069 (0.089)	0.400(0.343–0.474)***	0.007(-0.011–0.034)
DBP	TyG	0.422(0.033)***	0.138(0.036)***	0.259(0.061)***	0.394(0.325–0.471)***	0.036(0.016–0.067)**
	FBG	0.422(0.033)***	0.077(0.037)*	0.005(0.038)	0.394(0.325–0.471)***	0.000(-0.008–0.007)
	VAI	0.422(0.033)***	0.078(0.037)*	-0.233(0.076)**	0.394(0.325–0.471)***	-0.018(-0.048–-0.004)
	LAP	0.422(0.033)***	0.124(0.035)***	0.083(0.090)	0.394(0.325–0.471)***	0.010(-0.010–0.039)
MAP	TyG	0.452(0.033)***	0.136(0.037)***	0.251(0.060)***	0.424(0.360–0.499)***	0.034(0.014–0.066)*
	FBG	0.452(0.033)***	0.060(0.037)	0.013(0.037)	0.424(0.360–0.499)***	0.001(-0.007–0.007)
	VAI	0.452(0.033)***	0.071(0.037)	-0.211(0.075)**	0.424(0.360–0.499)***	-0.015(-0.048–-0.002)
	LAP	0.452(0.033)***	0.125(0.035)***	0.063(0.088)	0.424(0.360–0.499)***	0.008(-0.013–0.037)

c: total effect of the predictor on the outcome; β_1: effect of the predictor on the mediator; β_2: effect of the mediator on the outcome (adjusting for the predictor); c′: direct effect of the predictor on the outcome (adjusting for the mediator); β_{Ind}: indirect effect (mediation effect), calculated as $\beta_1 \times \beta_2$. Adjusted for age, obesity, smoking, alcohol use, and physical activity. BP, blood pressure; baPWV, brachial-ankle pulse wave velocity; SBP, systolic blood pressure; DBP, diastolic blood pressure; MAP, mean arterial pressure; TyG, triglyceride-glucose index; FBG, fasting blood glucose; VAI, visceral adiposity index; LAP, lipid accumulation product; SE, standard error; CI, confidence interval. $^{*}P<0.05$; $^{**}P<0.01$; $^{***}P<0.001$.

Table S6. Mediation effects of multiple metabolic factors on the relationship between adult BP and midlife baPWV in females.

Predictor	Mediator	c (SE)	β1 (SE)	β2 (SE)	c' (95%CI)	β_{Ind} (95%CI)
SBP	TyG	0.491(0.037)***	0.025(0.041)	0.017(0.073)	0.477(0.389–0.564)***	0.000(-0.006–0.011)
	FBG	0.491(0.037)***	0.131(0.042)**	0.093(0.042)*	0.477(0.389–0.564)***	0.012(-0.000–0.035)
	VAI	0.491(0.037)***	-0.014(0.042)	-0.021(0.090)	0.477(0.389–0.564)***	0.000(-0.013–0.011)
	LAP	0.491(0.037)***	0.011(0.037)	0.110 (0.100)	0.477(0.389–0.564)***	0.001(-0.012–0.021)
DBP	TyG	0.462(0.037)***	0.090 (0.041)*	-0.067(0.070)	0.448(0.345–0.550)***	-0.006(-0.023–0.007)
	FBG	0.462(0.037)***	0.126(0.042)**	0.120(0.041)**	0.448(0.345–0.550)***	0.015(0.001–0.042)
	VAI	0.462(0.037)***	0.013(0.041)	-0.001(0.091)	0.448(0.345–0.550)***	-0.000(-0.020–0.008)
	LAP	0.462(0.037)***	0.034(0.037)	0.145 (0.102)*	0.448(0.345–0.550)***	0.005(-0.010–0.022)
MAP	TyG	0.497(0.037)***	0.066(0.041)	-0.037(0.069)	0.482(0.384–0.575)***	-0.002(-0.015–0.009)
	FBG	0.497(0.037)***	0.134(0.042)**	0.105(0.041)*	0.482(0.384–0.575)***	0.014(0.001–0.040)
	VAI	0.497(0.037)***	0.002(0.041)	-0.003(0.090)	0.482(0.384–0.575)***	-0.000(-0.017–0.008)
	LAP	0.497(0.037)***	0.026(0.037)	0.125 (0.071)	0.482(0.384–0.575)***	0.003(-0.010–0.019)

c: total effect of the predictor on the outcome; β_1: effect of the predictor on the mediator; β_2: effect of the mediator on the outcome (adjusting for the predictor); c′: direct effect of the predictor on the outcome (adjusting for the mediator); β_{Ind}: indirect effect (mediation effect), calculated as $\beta_1 \times \beta_2$. Adjusted for age, obesity, smoking, alcohol use, and physical activity. BP, blood pressure; baPWV, brachial-ankle pulse wave velocity; SBP, systolic blood pressure; DBP, diastolic blood pressure; MAP, mean arterial pressure; TyG, triglyceride-glucose index; FBG, fasting blood glucose; VAI, visceral adiposity index; LAP, lipid accumulation product; SE, standard error; CI, confidence interval. $^{*}P<0.05$; $^{**}P<0.01$; $^{***}P<0.001$.

Table S7. Association between BP across the life course and adult baPWV among participants without hypertension, diabetes and cardiovascular events (n=1333).

Life stage	Exposure (BP measures)	Outcome (baPWV)		Relative importance
		β (95%CI)	*P* value	
Childhood†	SBP	3.46(2.37–4.55)	<0.001	16.73%
	DBP	3.34(2.13–4.55)	<0.001	12.60%
	MAP	4.24(2.94–5.53)	<0.001	15.70%
Adulthood††	SBP	7.52(6.48–8.56)	<0.001	40.63%
	DBP	7.21(6.20–8.22)	<0.001	38.78%
	MAP	7.55(6.59–8.51)	<0.001	41.42%
AUCt†††	SBP	2.49(1.34–3.63)	<0.001	11.70%
	DBP	2.51(0.68–4.34)	0.007	10.76%
	MAP	2.15(0.61–3.70)	0.006	11.72%

Relative importance was calculated using relative weight analysis, which decomposes the total R^2 from multiple regression models into weights that reflect the proportion of variance in the outcome variable (baPWV) attributable to each predictor (childhood BP, adulthood BP, and BP AUCt). This method accounts for multicollinearity among predictors by orthogonal transformation and is expressed as a percentage of the total explained variance. BP, blood pressure; baPWV, brachial-ankle pulse wave velocity; SBP, systolic blood pressure; DBP, diastolic blood pressure; MAP, mean arterial pressure; AUCt, area under the curve for total burden; CI, confidence interval. †Adjusted for age, sex, obesity, smoking, alcohol use, and physical activity. ††Adjusted for age, sex, obesity, smoking, alcohol use, physical activity and BP AUCt. †††Adjusted for age, sex, obesity, smoking, alcohol use, physical activity and adulthood BP.

Table S8. Mediation effects of multiple metabolic factors on the relationship between adult BP and midlife baPWV among participants without hypertension, diabetes and cardiovascular events (n=1333).

**Predictor**	**Mediator**	**c (SE)**	**β1 (SE)**	**β2 (SE)**	**c' (95%CI)**	**β_{Ind} (95%CI)**
SBP	TyG	0.426(0.026)***	0.078(0.029)**	0.190(0.044)***	0.407(0.351–0.463)***	0.015(0.003–0.030)*
	FBG	0.426(0.026)***	0.078(0.030)*	0.053(0.026)*	0.407(0.351–0.463)***	0.005(-0.004–0.012)
	VAI	0.426(0.026)***	0.029(0.030)	-0.155(0.057)**	0.407(0.351–0.463)***	-0.005(-0.021–0.004)
	LAP	0.426(0.026)***	0.068(0.028)*	0.073(0.065)	0.407(0.351–0.463)***	0.005(-0.004–0.021)
DBP	TyG	0.394(0.026)***	0.121(0.029)***	0.174(0.045)***	0.369(0.310–0.429)***	0.021(0.007–0.037)*
	FBG	0.394(0.026)***	0.098(0.030)**	0.054(0.027)*	0.369(0.310–0.429)***	0.005(-0.000–0.014)
	VAI	0.394(0.026)***	0.061(0.030)*	-0.166(0.058)**	0.369(0.310–0.429)***	-0.010(-0.029–-0.000)
	LAP	0.394(0.026)***	0.097(0.028)***	0.088(0.066)	0.369(0.310–0.429)***	0.009(-0.005–0.030)
MAP	TyG	0.437(0.026)***	0.111(0.029)***	0.176(0.044)***	0.414(0.353–0.472)***	0.020(0.006–0.035)*
	FBG	0.437(0.026)***	0.097(0.030)**	0.051(0.026)	0.414(0.353–0.472)***	0.005(-0.001–0.013)
	VAI	0.437(0.026)***	0.052(0.030)	-0.152(0.057)**	0.414(0.353–0.472)***	-0.008 (-0.027–0.001)
	LAP	0.437(0.026)***	0.092(0.028)**	0.072(0.065)	0.414(0.353–0.472)***	0.007(-0.006–0.026)

c: total effect of the predictor on the outcome; β_1: effect of the predictor on the mediator; β_2: effect of the mediator on the outcome (adjusting for the predictor); c′: direct effect of the predictor on the outcome (adjusting for the mediator); β_{Ind}: indirect effect (mediation effect), calculated as $\beta_1 \times \beta_2$. Adjusted for age, sex, obesity, smoking, alcohol use, and physical activity. BP, blood pressure; baPWV, brachial-ankle pulse wave velocity; SBP, systolic blood pressure; DBP, diastolic blood pressure; MAP, mean arterial pressure; TyG, triglyceride-glucose index; FBG, fasting blood glucose; VAI, visceral adiposity index; LAP, lipid accumulation product; SE, standard error; CI, confidence interval. $^{*}P<0.05$; $^{**}P<0.01$; $^{***}P<0.001$.

Table S9. Association between BP across the life course and adult baPWV among participants without medication use (n=1307).

Life stage	Exposure (BP measures)	Outcome (baPWV)		Relative importance
		β (95%CI)	*P* value	
Childhood†	SBP	3.31(2.23–4.39)	<0.001	16.24%
	DBP	3.13(1.95–4.32)	<0.001	14.33%
	MAP	4.01(2.73–5.29)	<0.001	16.81%
Adulthood††	SBP	7.15(6.08–8.22)	<0.001	36.29%
	DBP	6.71(5.68–7.74)	<0.001	33.25%
	MAP	7.12(6.13–8.11)	<0.001	36.42%
AUCt†††	SBP	2.40(1.25–3.54)	<0.001	20.58%
	DBP	2.72(0.89–4.55)	0.004	9.36%
	MAP	2.41(0.86–3.95)	0.002	10.43%

Relative importance was calculated using relative weight analysis, which decomposes the total R^2 from multiple regression models into weights that reflect the proportion of variance in the outcome variable (baPWV) attributable to each predictor (childhood BP, adulthood BP, and BP AUCt). This method accounts for multicollinearity among predictors by orthogonal transformation and is expressed as a percentage of the total explained variance. BP, blood pressure; baPWV, brachial-ankle pulse wave velocity; SBP, systolic blood pressure; DBP, diastolic blood pressure; MAP, mean arterial pressure; AUCt, area under the curve for total burden; CI, confidence interval. †Adjusted for age, sex, obesity, smoking, alcohol use, and physical activity. ††Adjusted for age, sex, obesity, smoking, alcohol use, physical activity and BP AUCt. †††Adjusted for age, sex, obesity, smoking, alcohol use, physical activity and adulthood BP.

Table S10. Mediation effects of multiple metabolic factors on the relationship between adult BP and midlife baPWV among participants without medication use (n=1307).

Predictor	Mediator	c (SE)	β1 (SE)	β2 (SE)	c' (95%CI)	$β_{Ind}$ (95%CI)
SBP	TyG	0.397(0.026)***	0.053(0.029)	0.195(0.044)***	0.378(0.325–0.429)***	0.010(0.003–0.024)*
	FBG	0.397(0.026)***	0.077(0.030)*	-0.150(0.058)**	0.378(0.325–0.429)***	0.007(0.001–0.018)
	VAI	0.397(0.026)***	0.038(0.029)	-0.155(0.057)**	0.378(0.325–0.429)***	-0.000(-0.010–0.007)
	LAP	0.397(0.026)***	0.378(0.026)***	0.057(0.066)	0.378(0.325–0.429)***	0.002(-0.003–0.012)
DBP	TyG	0.361(0.027)***	0.104(0.029)***	0.177(0.045)***	0.335(0.282–0.388)***	0.018(0.005–0.035)*
	FBG	0.361(0.027)***	0.108(0.030)***	0.084(0.027)**	0.335(0.282–0.388)***	0.009(0.002–0.023)
	VAI	0.361(0.027)***	0.039(0.030)	-0.163(0.059)**	0.335(0.282–0.388)***	-0.006(-0.021–0.001)
	LAP	0.361(0.027)***	0.070(0.029)*	0.074(0.067)	0.335(0.282–0.388)***	0.005(-0.005–0.022)
MAP	TyG	0.405(0.027)***	0.091(0.029)**	0.179(0.044)***	0.381(0.329–0.432)***	0.016(0.005–0.031)*
	FBG	0.405(0.027)***	0.103(0.031)***	0.081(0.026)**	0.381(0.329–0.432)***	0.008(0.001–0.022)
	VAI	0.405(0.027)***	0.026(0.031)	-0.149(0.058)*	0.381(0.329–0.432)***	-0.004(-0.016–0.002)
	LAP	0.405(0.027)***	0.062(0.029)*	0.060(0.066)	0.381(0.329–0.432)***	-0.004(-0.005–0.018)

c: total effect of the predictor on the outcome; $β_1$: effect of the predictor on the mediator; $β_2$: effect of the mediator on the outcome (adjusting for the predictor); c′: direct effect of the predictor on the outcome (adjusting for the mediator); $β_{Ind}$: indirect effect (mediation effect), calculated as $β_1 \times β_2$. Adjusted for age, sex, obesity, smoking, alcohol use, and physical activity. BP, blood pressure; baPWV, brachial-ankle pulse wave velocity; SBP, systolic blood pressure; DBP, diastolic blood pressure; MAP, mean arterial pressure; TyG, triglyceride-glucose index; FBG, fasting blood glucose; VAI, visceral adiposity index; LAP, lipid accumulation product; SE, standard error; CI, confidence interval. *P<0.05; **P<0.01; ***P<0.001.

Table S11. Association between BP across the life course and adult log-transformed baPWV.

Life stage	Exposure (BP measures)	Log-transformed baPWV		Relative importance
		**β (95%CI)**	***P* value**	
Childhood†	SBP	0.003(0.002–0.004)	<0.001	17.23%
	DBP	0.002(0.001–0.003)	<0.001	13.11%
	MAP	0.003(0.002–0.004)	<0.001	16.29%
Adulthood††	SBP	0.006(0.005–0.007)	<0.001	40.81%
	DBP	0.006(0.005–0.007)	<0.001	38.11%
	MAP	0.006(0.005–0.007)	<0.001	41.07%
AUCt†††	SBP	0.002(0.001–0.003)	<0.001	23.80%
	DBP	0.002(0.001–0.004)	0.002	10.85%
	MAP	0.002(0.001–0.003)	0.001	11.81%

Relative importance was calculated using relative weight analysis, which decomposes the total R^2 from multiple regression models into weights that reflect the proportion of variance in the outcome variable (baPWV) attributable to each predictor (childhood BP, adulthood BP, and BP AUCt). This method accounts for multicollinearity among predictors by orthogonal transformation and is expressed as a percentage of the total explained variance. BP, blood pressure; baPWV, brachial-ankle pulse wave velocity; SBP, systolic blood pressure; DBP: diastolic blood pressure; MAP, mean arterial pressure; AUCt, area under the curve for total burden; CI, confidence interval. †Adjusted for age, gender, obesity, smoking, alcohol use, and physical activity. ††Adjusted for age, gender, obesity, smoking, alcohol use, physical activity and BP AUCt. †††Adjusted for age, gender, obesity, smoking, alcohol use, physical activity and adulthood BP.

Table S12. Mediation effects of multiple metabolic factors on the relationship between adult BP and log-transformed baPWV in midlife.

Predictor	Mediator	c (SE)	β1 (SE)	β2 (SE)	c' (95%CI)	β_{Ind} (95%CI)
SBP	TyG	0.446(0.024)***	0.065(0.027)*	0.177(0.043)***	0.429(0.378–0.479)***	0.013(0.003–0.026)*
	FBG	0.446(0.024)***	0.025(0.029)	0.050(0.025)*	0.429(0.378–0.479)***	0.003(-0.001–0.010)
	VAI	0.446(0.024)***	0.071(0.029)**	-0.123(0.053)*	0.429(0.378–0.479)***	-0.003(-0.015–0.003)
	LAP	0.446(0.024)***	0.378(0.026)***	0.044(0.061)	0.429(0.378–0.479)***	0.003(-0.006–0.016)
DBP	TyG	0.419(0.025)***	0.114(0.025)***	0.159(0.043)***	0.398(0.343–0.453)***	0.018(0.006–0.032)**
	FBG	0.419(0.025)***	0.088(0.027)**	0.050 (0.026)	0.398(0.343–0.453)***	0.004(0.000–0.012)
	VAI	0.419(0.025)***	0.053(0.029)	-0.136(0.054)*	0.398(0.343–0.453)***	-0.007(-0.023–0.000)
	LAP	0.419(0.025)***	0.094(0.027)***	0.063(0.062)	0.398(0.343–0.453)***	0.006(-0.006–0.022)
MAP	TyG	0.459(0.024)***	0.105(0.028)***	0.162(0.043)***	0.439(0.385–0.492)***	0.017(0.005–0.031)**
	FBG	0.459(0.024)***	0.084(0.029)**	0.048(0.025)	0.439(0.385–0.492)***	0.004(-0.000–0.011)
	VAI	0.459(0.024)***	0.045(0.029)	-0.120(0.053)*	0.439(0.385–0.492)***	-0.005(-0.020–0.001)
	LAP	0.459(0.024)***	0.091(0.027)***	0.045(0.060)	0.439(0.385–0.492)***	0.004(-0.007–0.019)

c: total effect of the predictor on the outcome; β_1: effect of the predictor on the mediator; β_2: effect of the mediator on the outcome (adjusting for the predictor); c′: direct effect of the predictor on the outcome (adjusting for the mediator); β_{Ind}: indirect effect (mediation effect), calculated as $\beta_1 \times \beta_2$. Adjusted for age, sex, obesity, smoking, alcohol use, and physical activity. BP, blood pressure; baPWV, brachial-ankle pulse wave velocity; SBP, systolic blood pressure; DBP, diastolic blood pressure; MAP, mean arterial pressure; TyG, triglyceride-glucose index; FBG, fasting blood glucose; VAI, visceral adiposity index; LAP, lipid accumulation product; SE, standard error; CI, confidence interval. $^{*}P<0.05$; $^{**}P<0.01$; $^{***}P<0.001$.

Table S13. Association between BP across the life course and adult baPWV among participants with complete data after multiple imputation.

Life stage	Exposure (BP measures)	Outcome (baPWV)		Relative importance
		β (95%CI)	*P* value	
Childhood†	SBP	3.61(2.53–4.69)	<0.001	16.41%
	DBP	2.89(1.71–4.08)	<0.001	12.75%
	MAP	3.94(2.66–5.22)	<0.001	15.71%
Adulthood††	SBP	7.84(6.85–8.83)	<0.001	36.71%
	DBP	7.76(6.82–8.70)	<0.001	36.24%
	MAP	7.92(7.03–8.82)	<0.001	38.15%
AUCt†††	SBP	2.78(1.67–3.89)	<0.001	20.78%
	DBP	2.84(1.04–4.64)	0.002	11.72%
	MAP	2.84(1.04–4.64)	0.002	12.85%

Relative importance was calculated using relative weight analysis, which decomposes the total R^2 from multiple regression models into weights that reflect the proportion of variance in the outcome variable (baPWV) attributable to each predictor (childhood BP, adulthood BP, and BP AUCt). This method accounts for multicollinearity among predictors by orthogonal transformation and is expressed as a percentage of the total explained variance. BP, blood pressure; baPWV, brachial-ankle pulse wave velocity; SBP, systolic blood pressure; DBP, diastolic blood pressure; MAP, mean arterial pressure; AUCt, area under the curve for total burden; CI, confidence interval. †Adjusted for age, sex, obesity, smoking, alcohol use, and physical activity. ††Adjusted for age, sex, obesity, smoking, alcohol use, physical activity and BP AUCt. †††Adjusted for age, sex, obesity, smoking, alcohol use, physical activity and adulthood BP.

Table S14. Mediation effects of multiple metabolic factors on the relationship between adult BP and midlife baPWV among participants with complete data after multiple imputation.

Predictor	Mediator	c (SE)	β1 (SE)	β2 (SE)	c' (95%CI)	β_{Ind} (95%CI)
SBP	TyG	0.398(0.024)***	0.062(0.029)**	0.183(0.043)***	0.381(0.321–0.439)***	0.011(0.002–0.025)*
	FBG	0.398(0.024)***	0.062(0.028)**	0.047(0.026)*	0.381(0.321–0.439)***	0.003 (-0.001–0.010)
	VAI	0.398(0.024)***	0.014(0.028)	-0.155(0.054)*	0.381(0.321–0.439)***	-0.002 (-0.015–0.005)
	LAP	0.398(0.024)***	0.058(0.026)**	0.087(0.061)	0.381(0.321–0.439)***	0.005(-0.002–0.018)
DBP	TyG	0.397(0.025)***	0.118(0.027)***	0.158(0.043)**	0.373(0.313–0.435)***	0.019(0.006–0.035)*
	FBG	0.397(0.025)***	0.088(0.028)**	0.049 (0.026)	0.373(0.313–0.435)***	0.004(-0.000–0.013)
	VAI	0.397(0.025)***	0.055(0.028)	-0.161(0.054)**	0.373(0.313–0.435)***	-0.009(-0.024–-0.000)
	LAP	0.397(0.025)***	0.095(0.024)***	0.100 (0.062)	0.373(0.313–0.435)***	0.009(-0.002–0.027)
MAP	TyG	0.423(0.024)***	0.102(0.027)***	0.164(0.043)**	0.401(0.340–0.462)***	0.017(0.005–0.032)*
	FBG	0.423(0.024)***	0.082(0.029)**	0.047(0.025)	0.401(0.340–0.462)***	0.004(-0.001–0.012)
	VAI	0.423(0.024)***	0.041(0.029)	-0.149(0.053)**	0.401(0.340–0.462)***	-0.006(-0.020–0.001)
	LAP	0.423(0.024)***	0.085(0.027)	0.085(0.061)	0.401(0.340–0.462)***	0.007(-0.003–0.024)

c: total effect of the predictor on the outcome; β_1: effect of the predictor on the mediator; β_2: effect of the mediator on the outcome (adjusting for the predictor); c′: direct effect of the predictor on the outcome (adjusting for the mediator); β_{Ind}: indirect effect (mediation effect), calculated as $\beta_1 \times \beta_2$. Adjusted for age, sex, obesity, smoking, alcohol use, and physical activity. BP, blood pressure; baPWV, brachial-ankle pulse wave velocity; SBP, systolic blood pressure; DBP, diastolic blood pressure; MAP, mean arterial pressure; TyG, triglyceride-glucose index; FBG, fasting blood glucose; VAI, visceral adiposity index; LAP, lipid accumulation product; SE, standard error; CI, confidence interval. $^{*}P<0.05$; $^{**}P<0.01$; $^{***}P<0.001$.

Table S15. Comparison of baseline demographic and clinical characteristics between participants who remained in the final analysis and those who dropped out during follow-up.

Variable	Retained (n=1,448)	Drop-out (n=1,843)	*P* value
**Childhood (1987)**			
Age, years	12(9–14)	12(10–14)	0.196
Boys, n(%)	809(55.87)	1077(58.45)	0.168
BMI, kg/m^2	16.2(14.5–18.2)	16.4(15.0–18.5)	0.128
Bust, cm	65.2(60.0–72.0)	63.9(58.0–70.5)	0.094
Heart rate, beats/min	78.0(72.0–84.0)	78.0(72.0–84.0)	0.071
SBP, mmHg	102.0(96.0–110.0)	105.3(98.0–112.0)	0.149
DBP, mmHg	64.0(60.0–70.7)	65.0(60.0–71.3)	0.530
MAP, mmHg	71.1(71.5–83.3)	71.9(70.9–84.6)	0.723

Values are expressed as median (IQR) if non-normal distribution. BMI, body mass index; SBP, systolic blood pressure; DBP, diastolic blood pressure; MAP, mean arterial pressure.

Table S16. Normality assessment of continuous variables using the Shapiro–Wilk test.

Variable	Numbers	W	V	Z score	*P* value
**Childhood (1987)**					
Age, years	1,448	0.98356	14.534	6.726	<0.001
SBP, mmHg	1,448	0.98957	9.218	5.582	<0.001
DBP, mmHg	1,448	0.99273	6.426	4.675	<0.001
MAP, mmHg	1,448	0.99439	4.955	4.022	<0.001
**Adulthood (2017)**					
Age, years	1,448	0.98356	14.534	6.726	<0.001
SBP, mmHg	1,448	0.97842	19.07	7.409	<0.001
DBP, mmHg	1,448	0.98105	16.744	7.082	<0.001
MAP, mmHg	1,448	0.97911	18.465	7.328	<0.001
FBG, mmol/L	1,397	0.47159	451.982	15.343	<0.001
LDL-C, mmol/L	1,397	0.98276	14.748	6.754	<0.001
HDL-C, mmol/L	1,397	0.96348	31.241	8.637	<0.001
TyG, mmol/L	1,397	0.96815	27.241	8.294	<0.001
LAP	1,395	0.70995	247.777	13.833	<0.001
VAI	1,394	0.54474	388.653	14.963	<0.001
AIP	1,397	0.52325	407.796	15.085	<0.001
SUA, umol/L	1,397	0.98569	12.241	6.286	<0.001
uACR, mg/g	1,347	0.15295	700.854	16.420	<0.001
baPWV, cm/s	1,448	0.96322	32.506	8.749	<0.001
**Long-term burden**					
SBP AUCt, mmHg	1,448	0.99474	4.649	3.862	<0.001
DBP AUCt, mmHg	1,418	0.96812	27.645	8.335	<0.001
MAP AUCt, mmHg	1,418	0.96656	28.992	8.455	<0.001

The Shapiro–Wilk test was used to evaluate the normality of continuous variables. AUCt, area under the curve for total burden; SBP, systolic blood pressure; DBP, diastolic blood pressure; MAP, mean arterial pressure; FBG, fasting blood glucose; LDL-C, low-density lipoprotein cholesterol; HDL-C, high-density lipoprotein cholesterol; TyG, triglyceride–glucose index; LAP, lipid accumulation product; VAI, visceral adiposity index; AIP, atherogenic index of plasma; SUA, serum uric acid; uACR, urinary albumin-to-creatinine ratio; baPWV, brachial-ankle pulse wave velocity.

Table S17. Assessment of collinearity among covariates.

Covariates	SBP Model	DBP Model	MAP Model
Childhood BP	1.07	1.02	1.05
Adult BP	1.13	1.11	1.14
BP AUCt	1.01	1.01	1.01
Age	1.03	1.01	1.02
Sex	1.52	1.52	1.52
Obesity	1.03	1.03	1.03
Smoking	1.47	1.47	1.47
Drinking	1.15	1.15	1.15
Physical activity	1.01	1.01	1.01

Collinearity among variables in the model was assessed using the standardized variance inflation factor (VIF). A VIF value < 2 was considered indicative of low multicollinearity, suggesting minimal influence on the stability and reliability of the model estimates. BP, blood pressure; AUCt, area under the curve for total burden; SBP, systolic blood pressure; DBP, diastolic blood pressure; MAP, mean arterial pressure.

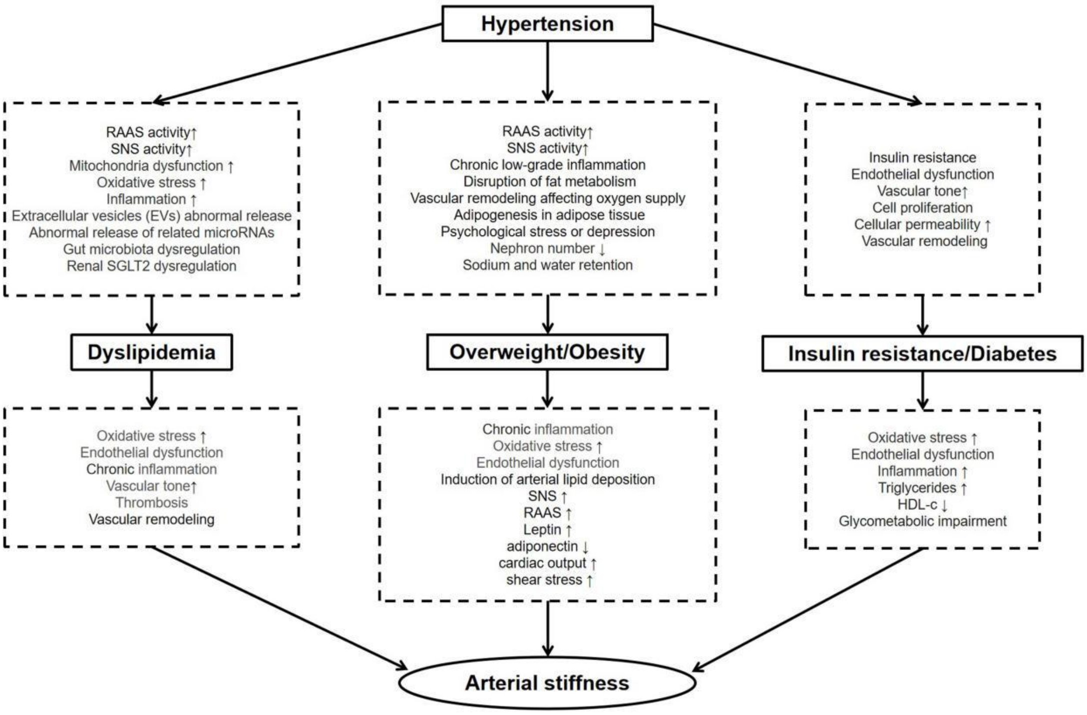

Figure S1. Potential Biological mechanisms underlying the relationships among hypertension, obesity, dyslipidemia, insulin resistance and arterial stiffness. RAAS, renin–angiotensin–aldosterone system; SNS, sympathetic nervous system; EVs, extracellular vesicles; SGLT2, sodium-glucose co-transporter 2.

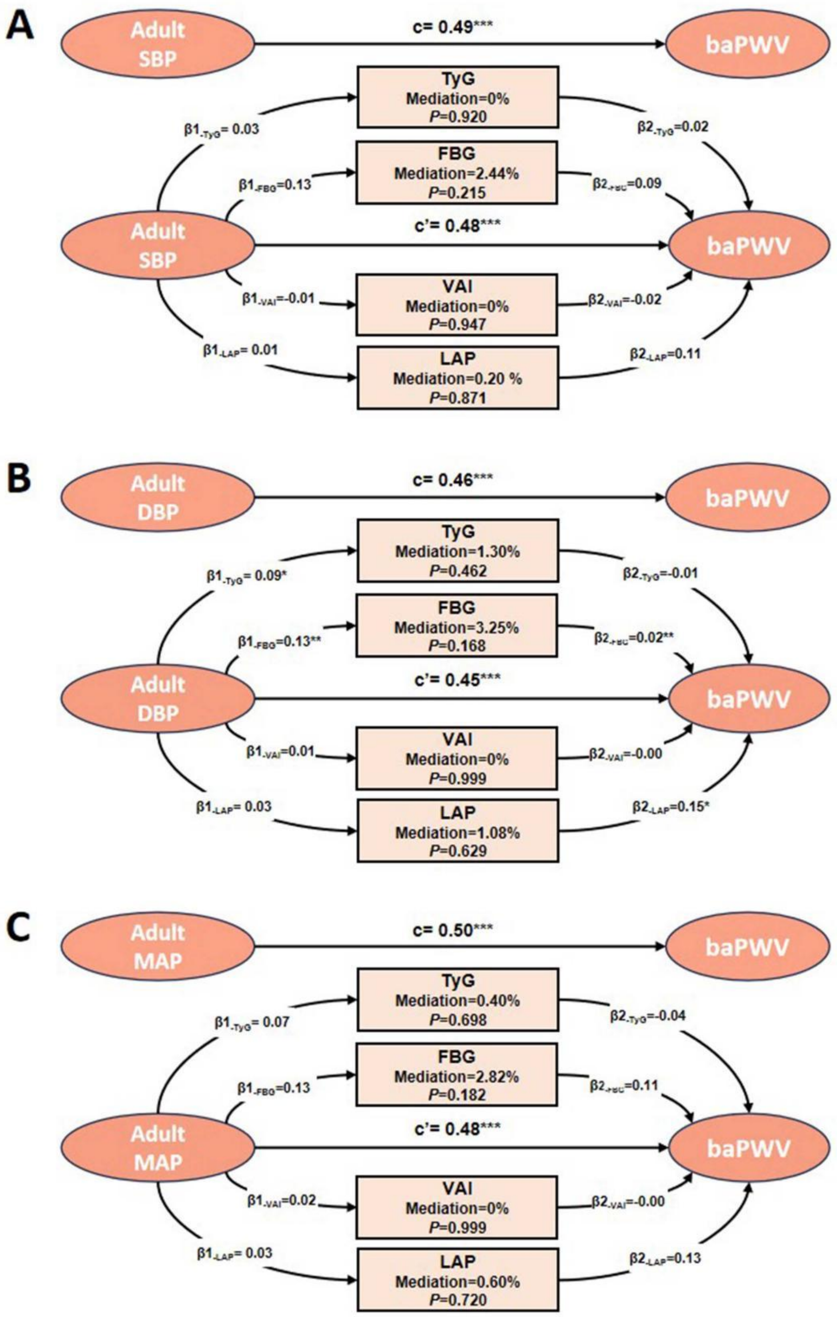

Figure S2. Mediation effects of multiple metabolic factors on the association between adult BP and midlife baPWV in females. BP, blood pressure; baPWV, brachial-ankle pulse wave velocity; SBP, systolic blood pressure; DBP, diastolic blood pressure; MAP, mean arterial ressure; FBG, fasting blood glucose; TyG, index triglyceride–glucose index; VAI, visceral adiposity index. LAP, lipid accumulation product. Adjusted for age, obesity, smoking, alcohol use, physical activity. c: total effect of the predictor on the outcome; β_1: effect of the predictor on the mediator; β_2: effect of the mediator on the outcome (adjusting for the predictor); c′: direct effect of the predictor on the outcome (adjusting for the mediator). $^{*}P < 0.05$; $^{**}P < 0.01$; $^{***}P < 0.001$.

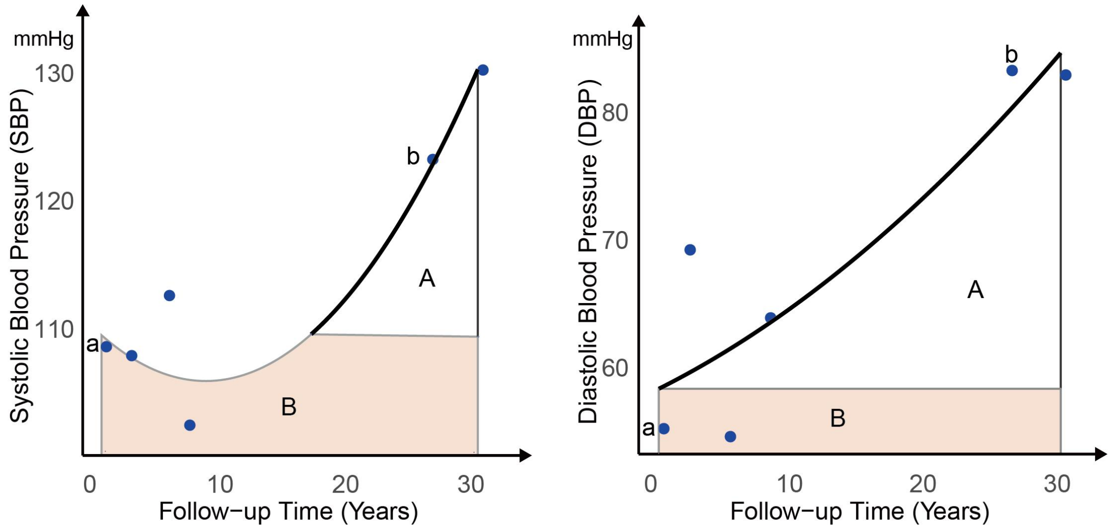

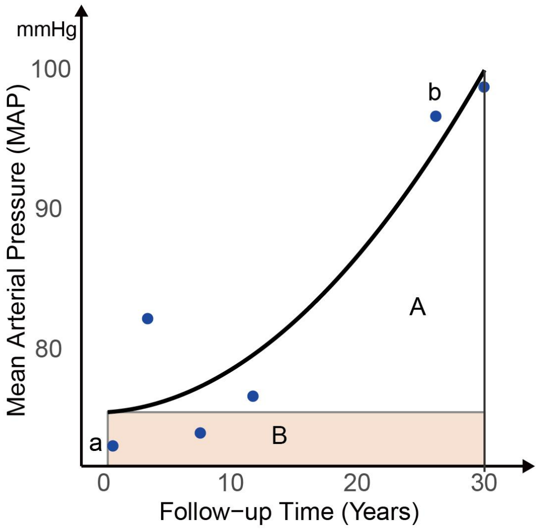

Example for ID: 19169:	
Point a	Childhood BP
Point b	Adulthood BP
Shape A	Increasmental AUC
Shape (A+B)	Total AUC

Figure S3. Schematic diagram illustrating the calculation of long-term cumulative blood pressure exposure using the area under the curve (AUC). BP, blood pressure. AUC, area under the curve.

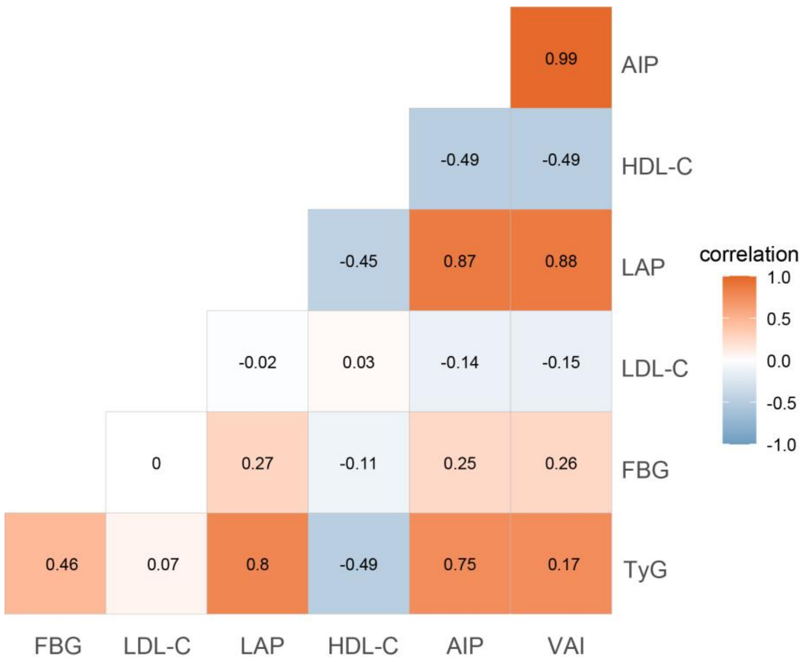

Figure S4. Correlations among multiple metabolic factors. AIP, atherosclerotic index of plasma; HDL-C, high-density lipoprotein cholesterol; LAP, lipid accumulation product; LDL-C, low-density lipoprotein cholesterol; FBG, fasting blood glucose; TyG, triglyceride-glucose index; VAI, visceral adiposity index.

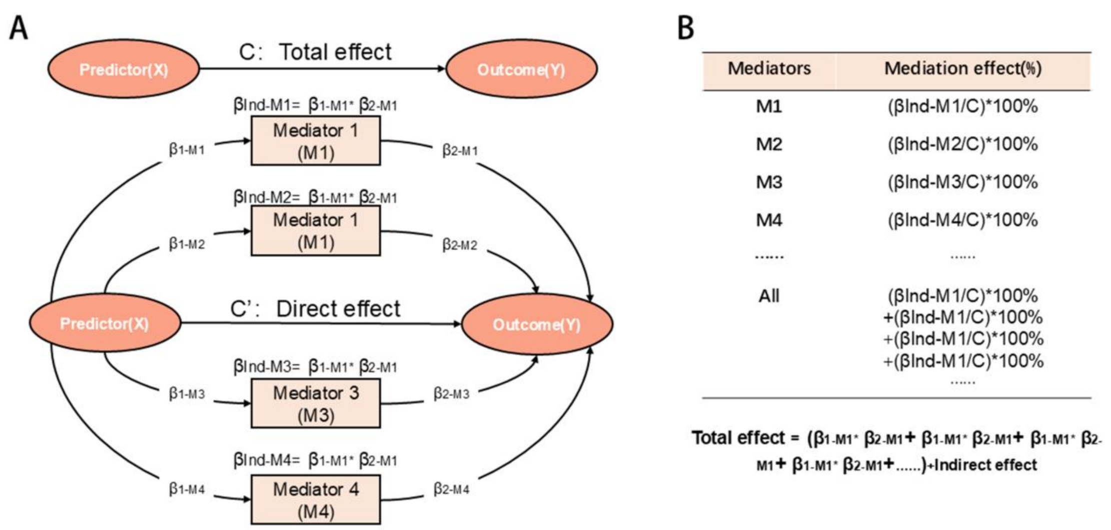

Figure S5. Schematic representation of the framework for multiple parallel mediation analysis. A: the framework of multiple parallel mediation; B: calculation of the proportion of mediation effects.
